# MEMS Young’s Modulus and Step Height Measurements With Round Robin Results

**DOI:** 10.6028/jres.115.023

**Published:** 2010-10-01

**Authors:** Janet Marshall, Richard A. Allen, Craig D. McGray, Jon Geist

**Affiliations:** Semiconductor Electronics Division, National Institute of Standards and Technology, Gaithersburg, MD 20899-8120

**Keywords:** interferometry, microelectromechanical systems, round robin, standard test methods, step height, test structure, vibrometry, Young’s modulus

## Abstract

This paper presents the results of a microelectromechanical systems (MEMS) Young’s modulus and step height round robin experiment, completed in April 2009, which compares Young’s modulus and step height measurement results at a number of laboratories. The purpose of the round robin was to provide data for the precision and bias statements of two \ related Semiconductor Equipment and Materials International (SEMI) standard test methods for MEMS. The technical basis for the test methods on Young’s modulus and step height measurements are also provided in this paper.

Using the same test method, the goal of the round robin was to assess the repeatability of measurements at one laboratory, by the same operator, with the same equipment, in the shortest practical period of time as well as the reproducibility of measurements with independent data sets from unique combinations of measurement setups and researchers. Both the repeatability and reproducibility measurements were done on random test structures made of the same homogeneous material.

The average repeatability Young’s modulus value (as obtained from resonating oxide cantilevers) was 64.2 GPa with 95 % limits of ± 10.3 % and an average combined standard uncertainty value of 3.1 GPa. The average reproducibility Young’s modulus value was 62.8 GPa with 95 % limits of ± 11.0 % and an average combined standard uncertainty value of 3.0 GPa.

The average repeatability step height value (for a metal2-over-poly1 step from active area to field oxide) was 0.477 μm with 95 % limits of 7.9 % and an average combined standard uncertainty value of 0.014 μm. The average reproducibility step height value was 0.481 μm with 95 % limits of ± 6.2 % and an average combined standard uncertainty value of 0.014 μm.

In summary, this paper demonstrates that a reliable methodology can be used to measure Young’s modulus and step height. Furthermore, a micro and nano technology (MNT) 5-in-1 standard reference material (SRM) can be used by industry to compare their in-house measurements using this methodology with NIST measurements thereby validating their use of the documentary standards.

**Table:** 

List of Terms	
*Cantilever*	a test structure that consists of a freestanding beam that is fixed at one end^1^
*fixed-fixed beam*	a test structure that consists of a freestanding beam that is fixed at both ends^1^
*in-plane length (or deflection) measurement*	
	the experimental determination of the straight-line distance between two transitional edges in a MEMS device^1^
*Interferometer*	a non-contact optical instrument used to obtain topographical 3-D data sets^1^
*residual strain*	in a MEMS process, the amount of deformation (or displacement) per unit length constrained within the structural layer of interest after fabrication yet before the constraint of the sacrificial layer (or substrate) is removed (in whole or in part)^1^
*residual stress*	the remaining forces per unit area within the structural layer of interest after the original cause(s) during fabrication have been removed yet before the constraint of the sacrificial layer (or substrate) is removed (in whole or in part)^2^
*step height*	the distance in the *z*-direction that an initial, flat, processed surface (or platform) is to a final, flat, processed surface (or platform)^2^
*(residual) strain gradient*	a through-thickness variation (of the residual strain) in the structural layer of interest before it is released^1^
(*residual*) stress *gradient*	a through-thickness variation (of the residual stress) in the structural layer of interest before it is released^2^
*test structure*	a component (such as, a fixed-fixed beam or cantilever) that is used to extract information (such as, the residual strain or the strain gradient of a layer) about a fabrication process^1^
*Thickness*	the height in the *z*-direction of one or more designated thin-film layers^2^
*Vibrometer*	an instrument for non-contact measurements of surface motion^2^
*Young’s modulus*	a parameter indicative of material stiffness that is equal to the stress divided by the strain when the material is loaded in uniaxial tension, assuming the strain is small enough such that it does not irreversibly deform the material^2^

**Table:** 

List of Symbols	
*For Young’s modulus measurements and calculations:*	
*μ*	= viscosity of the ambient surrounding the cantilever. [SEMI MS4]
*ρ*	= density of the thin film layer. [SEMI MS4]
*E*	= calculated Young’s modulus value of the thin film layer. [SEMI MS4]
*E*_clamped_	= calculated Young’s modulus value obtained from the average resonance frequency of a fixed-fixed beam assuming clamped-clamped boundary conditions. [SEMI MS4]
*E*_init_	= initial estimate for the Young’s modulus value of the thin film layer. [SEMI MS4]
*E*_simple_	= calculated Young’s modulus value obtained from the average resonance frequency of a fixed-fixed beam assuming simply supported boundary conditions at both supports. [SEMI MS4]
*f*_can_	= average undamped resonance frequency of the cantilever. [SEMI MS4]
*f*_ffb_	= average resonance frequency of the fixed-fixed beam. [SEMI MS4]
*L*_can_	= suspended cantilever length. [SEMI MS4]
*L*_ffb_	= suspended fixed-fixed beam length. [SEMI MS4]
*Q*	= oscillatory quality factor of the cantilever. [SEMI MS4]
*t*	= thickness of the thin film layer. [SEMI MS4]
*W*_can_	= suspended cantilever width. [SEMI MS4]
*For combined standard uncertainty calculations of Young’s modulus measurements:*	
*σ_μ_*	= one sigma uncertainty of the value of *μ*. [SEMI MS4]
*σ_ρ_*	= one sigma uncertainty of the value of *ρ*. [SEMI MS4]
*σ*_freq_	= one sigma uncertainty of the value of *f*_can_. [SEMI MS4]
*σ_L_*	= one sigma uncertainty of the value of *L*_can_. [SEMI MS4]
*σ*_thick_	= one sigma uncertainty of the value of *t*. [SEMI MS4]
*σ_W_*	= one sigma uncertainty of the value of *W*_can_. [SEMI MS4]
*E*_max_	= maximum Young’s modulus value as determined in an uncertainty calculation. [SEMI MS4]
*E*_min_	= minimum Young’s modulus value as determined in an uncertainty calculation. [SEMI MS4]
*f*_resol_	= frequency resolution for the given set of measurement conditions. [SEMI MS4]
*p*_diff_	= estimated percent difference between the damped and undamped resonance frequency of the cantilever. [SEMI MS4]
*u_ρ_*	= component in the combined standard uncertainty calculation for Young’s modulus that is due to the uncertainty of *ρ*. [SEMI MS4]
*u*_c_	= combined standard uncertainty value (that is, the estimated standard deviation of the result). [SEMI MS4]
*u*_c_*_E_*	= the combined standard uncertainty for a Young’s modulus measurement.
*u*_damp_	= component in the combined standard uncertainty calculation for Young’s modulus that is due to damping. [SEMI MS4]
*u_E_*	= the standard uncertainty for a Young’s modulus measurement as obtained from a fixed-fixed beam.
*u*_freq_	= component in the combined standard uncertainty calculation for Young’s modulus that is due to the measurement uncertainty of f_can_. [SEMI MS4]
$u_{f_{\text{resol}}}$	= component in the combined standard uncertainty calculation for Young’s modulus that is due to *f*_resol_. [SEMI MS4]
*u_L_*	= component in the combined standard uncertainty calculation for Young’s modulus that is due to the measurement uncertainty of *L*_can_. [SEMI MS4]
*u*_thick_	= component in the combined standard uncertainty calculation for Young’s modulus that is due to the measurement uncertainty of *t*. [SEMI MS4]
*For calibration of step height measurements:*	
*σ_cert_*	= the one sigma uncertainty of the calibrated physical step height used for calibration.
*cert*	= the certified value of the double-sided physical step height used for calibration. [SEMI MS2]
*z*_drift_	= the calibrated positive difference between the average of the six calibration measurements taken before the data session (at the same location on the physical step height used for calibration) and the average of the six calibration measurements taken after the data session (at the same location on the physical step height used for calibration). [SEMI MS2]
*z*_perc_	= over the instrument’s total scan range, the maximum percent deviation from linearity, as quoted by the instrument manufacturer (typically less than 3 %). [SEMI MS2]
*z*_repeat_	= the maximum of two calibrated values; one of which is the positive calibrated difference between the minimum and maximum values of the six calibration measurements taken before the data session (at the same location on the physical step height used for calibration) and the other is the positive calibrated difference between the minimum and maximum values of the six calibration measurements taken after the data session (at the same location on the physical step height used for calibration). [SEMI MS2]
$\bar{z}$	= calibrated average of the twelve calibration measurements taken before and after the data session at the same location on the physical step height used for calibration. [SEMI MS2]
${\bar{z}}_{6}$	= the calibrated average of the six calibration measurements from which *z*repeat is found. [SEMI MS2]
*For step height measurements and calculations:*	
*σ_platNXt_*	= the calibrated standard deviation of the data from trace “*t*” on *platNX*. [SEMI MS2]
*σ*_rough_*_NX_*	= the calibrated surface roughness of *platNX* measured as the smallest of all the values obtained for *σ_platNXt_*; however, if the surfaces of the platforms (including the reference platform) all have identical compositions, then it is measured as the smallest of all the standard deviation values obtained from data traces “a,” “b,” and “c” along these platforms. [SEMI MS2]
*platNrD*	= the average of the calibrated reference platform height measurements taken from multiple data traces on one step height test structure, where *N* is the test structure number (“1,” “2,” “3,” etc.), r indicates it is from a reference platform, and *D* directionally indicates which reference platform (using the compass indicators “N,” “S,” “E,” or “W” where “N” refers to the reference platform designed closest to the top of the chip). [SEMI MS2]
*platNrDt*	= a calibrated reference platform height measurement from one data trace, where *N* is the test structure number (“1,” “2,” “3,” etc.), r indicates it is from a reference platform, *D* directionally indicates which reference platform (using the compass indicators “N,” “S,” “E,” or “W” where “N” refers to the reference platform designed closest to the top of the chip), and *t* is the data trace (“a,” “b,” “c,” etc.) being examined. [SEMI MS2]
*platNX*	= the calibrated platform height measurement, where *N* is the test structure number (“1,” “2,” “3,” etc.) and *X* is the capital letter (or “r” is used if it is the reference platform) associated with the platform (“A,” “B,” “C,” etc.) as lettered starting with “A” for the platform closest to *platNrW* or *platNrS*. [SEMI MS2]
*platNXt*	= a calibrated platform height measurement from one data trace, where *N* is the test structure number (“1,” “2,” “3,” etc.), *X* is the capital letter associated with the platform (“A,” “B,” “C,” etc.) as lettered starting with “A” for the platform closest to *platNrW* or *platNrS*, and *t* is the data trace (“a,” “b,” “c,” etc.) being examined. [SEMI MS2]
*stepN_X_M_Y_*	= the calibrated step height measurement taken from two different step height test structures (*N* and *M*) on the same test chip, which is equal to the final platform height minus the initial platform height, where the step is from the initial platform to the final platform and where *X* is the capital letter associated with the initial platform from test structure number *N*, and *Y* is the capital letter associated with the final platform from test structure number *M*. [SEMI MS2]
*stepN_XY_*	= the average of the calibrated step height measurements taken from multiple data traces on one step height test structure, where *N* is the number associated with the test structure, *X* is the capital letter associated with the initial platform (or “r” is used if it is the reference platform), *Y* is the capital letter associated with the final platform (or “r” is used if it is the reference platform), and the step is from the initial platform to the final platform. [SEMI MS2]
*stepN_XYt_*	= a calibrated step height measurement from one data trace on one step height test structure, where *N* is the number associated with the test structure, *X* is the capital letter associated with the initial platform (or “r” is used if it is the reference platform), *Y* is the capital letter associated with the final platform (or “r” is used if it is the reference platform), *t* is the data trace (“a,” “b,” “c,” etc.) being examined, and the step is from the initial platform to the final platform. [SEMI MS2]
*For combined standard uncertainty calculations of step height measurements:*	
*u*_c_	= the combined standard uncertainty value (i.e., the estimated standard deviation of the result). [SEMI MS2]
*u_cert_*	= the component in the combined standard uncertainty calculation that is due to the uncertainty of the value of the physical step height used for calibration. [SEMI MS2]
*u*_cSH_	= the combined standard uncertainty or a step height measurement.
*u*_drift_	= the uncertainty of a measurement due to the amount of drift during the data session.
*u*_linear_	= the uncertainty of a measurement due to the deviation from linearity of the data scan. [SEMI MS2]
*u_LplatNX_*	= the component in the combined standard uncertainty calculation for platform height measurements that is due to the measurement uncertainty across the length of *platNX*, where the length is measured perpendicular to the edge of the step. [SEMI MS2]
*u_L_*_step_	= the component in the combined standard uncertainty calculation for step height measurements that is due to the measurement uncertainty of the step height across the length of the step, where the length is measured perpendicular to the edge of the step. [SEMI MS2]
*u_platNX_*	= the component in the combined standard uncertainty calculation or step height measurements obtained from two step height test structures that is due to the uncertainty of the platform height measurement for *platNX*. [SEMI MS2]
*u*_repeat_	= the uncertainty of a measurement due to the repeatability of a measurement. [SEMI MS2]
*u_WplatNX_*	= the component in the combined standard uncertainty calculation for platform height measurements that is due to the measurement uncertainty across the width of *platNX*, where the width is measured parallel to the edge of the step. [SEMI MS2]
*u_W_*_step_	= the component in the combined standard uncertainty calculation for step height measurements that is due to the measurement uncertainty of the step height across the width of the step, where the width is measured parallel to the edge of the step. [SEMI MS2]
*For residual stress and stress gradient calculations:*	
*ε*_r_	= residual strain of the thin film layer. [SEMI MS4]
*σ*_g_	= stress gradient of the thin film layer. [SEMI MS4]
*σ*_gmax_	= maximum stress gradient value as determined in an uncertainty calculation. [SEMI MS4]
*σ*_gmin_	= minimum stress gradient value as determined in an uncertainty calculation. [SEMI MS4]
*σ*_r_	= residual stress of the thin film layer. [SEMI MS4]
*σ*_rmax_	= maximum residual stress value as determined in an uncertainty calculation. [SEMI MS4]
*σ*_rmin_	= minimum residual stress value as determined in an uncertainty calculation. [SEMI MS4]
*s*_g_	= strain gradient of the thin film layer. [SEMI MS4]
*u_εr_*_(_*_σr_*_)_	= component in the combined standard uncertainty calculation for residual stress that is due to the measurement uncertainty of *ε*_r_. [SEMI MS4]
*u_cεr_*	= combined standard uncertainty value for residual strain.
*u_cσg_*	= combined standard uncertainty value for stress gradient.
*u_cσr_*	= combined standard uncertainty value for residual stress.
*u_csg_*	= combined standard uncertainty value for strain gradient.
*u_E_*_(_*_σg_*_)_	= component in the combined standard uncertainty calculation for stress gradient that is due to the measurement uncertainty of *E*. [SEMI MS4]
*u_E_*_(_*_σr_*_)_	= component in the combined standard uncertainty calculation for residual stress that is due to the measurement uncertainty of *E*. [SEMI MS4]
*u_sg_*_(_*_σg_*_)_	= component in the combined standard uncertainty calculation for stress gradient that is due to the measurement uncertainty of *s*_g_. [SEMI MS4]
*For round robin measurements:*	
*n*	= the number of reproducibility or repeatability measurements.
*u*_cave_	= the average combined standard uncertainty value for the reproducibility or repeatability measurements. It is equal to the sum of the *u*_c_ values divided by *n*.

## 1. Introduction

Microelectromechanical systems (MEMS) foundaries are emerging from the economic downturn in a strong position [1]. Applications for MEMS demand robust reliability [2], which necessitates the consideration of reliability at the earliest stages of product development [3]. The U.S. Measurement System (USMS) stated that there are insufficient measurement data and measurement methods to generate that data to adequately characterize a MEMS device and its dependence on fabrication processes [4]. In response to this, SEMI has published two MEMS standard test methods; one for measuring Young’s modulus [5] and one for measuring step heights [6]. This paper presents the technical basis for the standards and the data from a MEMS Young’s modulus and step height round robin experiment, which was used to validate these standards. These standards are expected to facilitate commerce in MEMS technologies and improve manufacturing yields by decreasing interlaboratory differences in these measurements.

The first MEMS standards were approved by ASTM in 2002 then validated in 2005 [7–9] with round robin precision and bias data. These ASTM test methods are used for measuring in-plane lengths or deflections, residual strain, and strain gradient. They employ a non-contact measurement approach using an optical interferometer.^3^

The SEMI test method for measuring Young’s modulus that is reported in this paper uses an optical vibrometer, stroboscopic interferometer, or comparable instrument, and the SEMI test method for step height measurements also reported in this paper uses an optical interferometer or comparable instrument. Therefore, a stroboscopic interferometer can be used for all five standard test methods for MEMS (the three ASTM test methods and the two SEMI test methods).

**Fig. 1:**
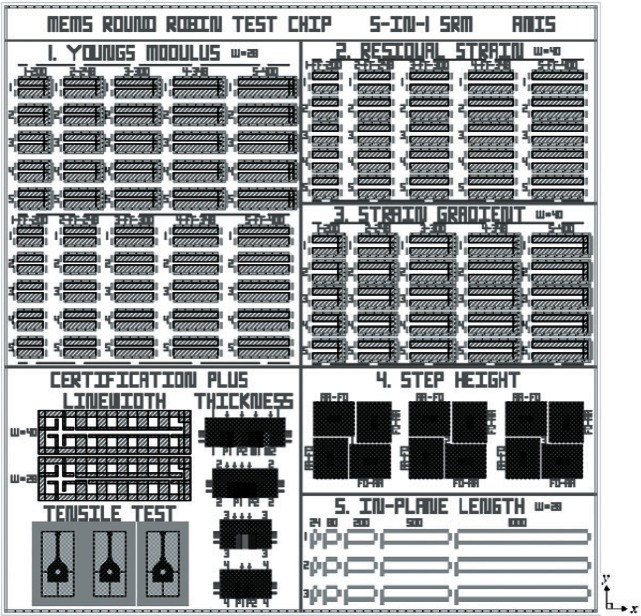
The MEMS Young’s Modulus and Step Height Round Robin Test Chip.

Test structures for use with all five standard test methods are incorporated on the MEMS Young’s Modulus and Step Height Round Robin Test Chip, which is shown in Fig. 1. This chip is also the prototype for a micro and nano technology (MNT) 5-in-1 standard reference material (SRM) [10]. Using the MNT 5-in-1 SRM, companies will be able to compare their in-house measurements taken on the SRM with NIST measurements thereby validating their use of the documentary standards.

**Fig. 2:**
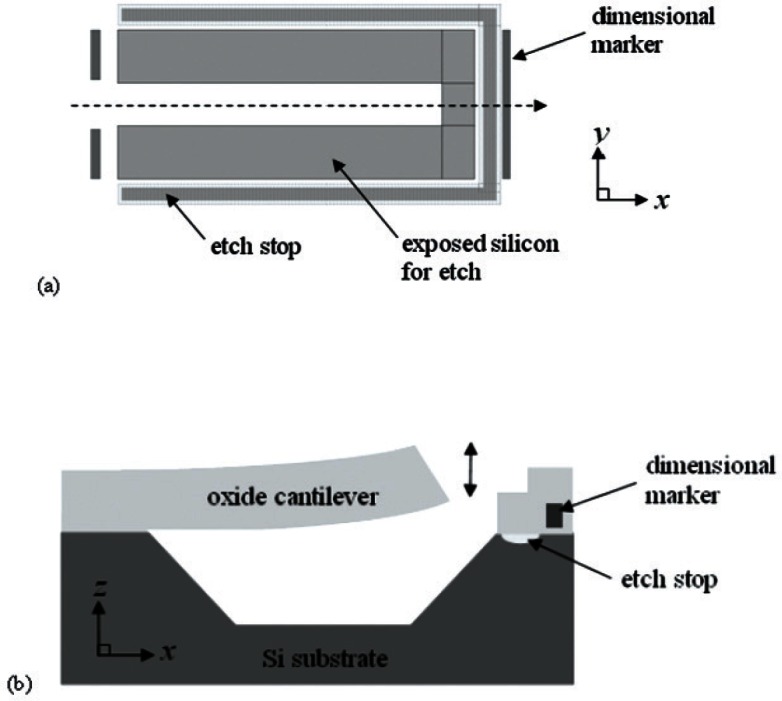
For a cantilever test structure on the test chip shown in Fig. 1 (a) a design rendition and (b) a cross section. In Fig. 2(a), the central structure (silicon dioxide and silicon nitride atop the silicon substrate) will become an all oxide cantilever after the silicon nitride is removed and the exposed raw silicon on three of the structures sides and beneath it is etched, as shown in Fig. 2(b). The dimensional markers are typically made of polysilicon or metal encapsulated in oxide and can be used to obtain a more accurate measurement of the cantilever length after the post-processing etch.

The first test method (SEMI MS4) [5] to be presented in this paper is on Young’s modulus measurements of thin, reflecting films. As presented in [11], there are many Young’s modulus methods with values that have been reported in the literature. The method reported here is based on the textbook treatment [12] of the average resonance frequency of a single-layered cantilever oscillating out-of-plane (recommended and shown in Fig. 2) or based on the average resonance frequency of a single layered fixed-fixed beam oscillating out-of-plane. The resonance frequency method was chosen due to a) its wide acceptance in the community, b) the involvement of simple, non-contact, non-destructive measurements, and c) its ability to obtain measurements from test structures on a single test chip alongside other test structures. A piezoelectric transducer (PZT) is used to create the out-of-plane excitations; however, a PZT is not the only means available for excitation (for example, thermal excitation [13–15] is possible). Given the value for Young’s modulus, residual stress and stress gradient calculations are possible.

Young’s modulus measurements are an aid in the design and fabrication of MEMS devices and integrated circuits (ICs). For example, high values of residual stress can lead to failure mechanisms in ICs such as electromigration, stress migration, and delamination. So, methods for its characterization are of interest for IC process development and monitoring in order to improve the yield in complementary metal oxide semiconductor (CMOS) fabrication processes [16].

**Fig. 3:**
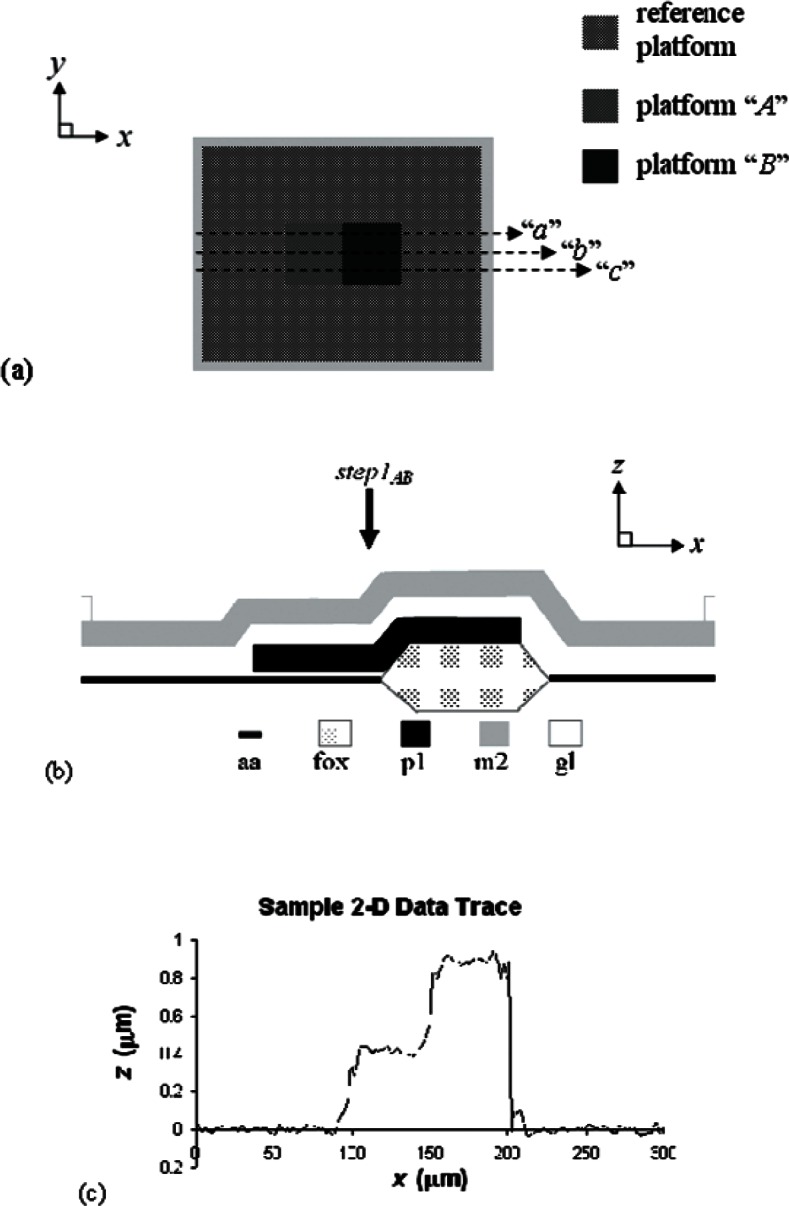
For the step height test structure, on the test chip shown in Fig. 1, with a 0° orientation (a) a design rendition showing 2-D data traces “a,” “b,” and “c,” (b) its cross section, (where aa indicates active area, fox indicates field oxide, pl indicates polyl, m2 indicates metal2, and gl indicates glass), and (c) a sample 2-D data trace.

The second test method (SEMI MS2) [6] to be presented in this paper is for step height measurements of thin films. A step height test structure, such as shown in Fig. 3(a) with its cross section given in Fig. 3(b), is used for these measurements. Multiple 2-D data traces along the top of this test structure, such as traces “a,” “b,” and “c” in Fig. 3(a), are obtained. A sample 2-D data trace is given in Fig. 3(c). Data averages and standard deviations from the 2-D data traces along the pertinent platforms are recorded.

For data obtained from one step height test structure, the difference in the platform heights involved in the step for each 2-D data trace is calculated. The step height, *stepN_XY_*, [or *step1_AB_* as shown in Fig. 3(b)] is the average of these values from the different 2-D data traces.

Step height measurements can also be obtained from two step height test structures. The calculations are slightly different, and this approach is not recommended due to higher resulting combined standard uncertainty values [17].

Step height measurements can be used to determine thin film thickness values [18,19]. Thickness measurements are an aid in the design and fabrication of MEMS devices and can be used to obtain thin film materials parameters, such as Young’s modulus [5,16].

The SEMI test methods for Young’s modulus and step height measurements were used in the MEMS Young’s modulus and step height round robin experiment to determine the repeatability of a measurement as well as to see if independent laboratories could reproduce these measurements without introducing a bias.

For the round robin, test chips were fabricated on the same processing run. One test chip (the design of which is shown in Fig. 1) was delivered to each laboratory, and measurements were taken using the procedures in the SEMI test methods. The data from these measurements were analyzed using a NIST web-based program [10] that also verifies the data. Five laboratories (including NIST) participated in the round robin, resulting in eight independent data sets (due to different equipment setups) for Young’s modulus and seven independent data sets (due to different equipment setups) for step heights. The results from this round robin are reported in this paper.

Section 2 presents the packaged Round Robin Test Chip. Section 3 presents the technical basis for the Young’s modulus and step height standard test methods. Section 4 presents the uncertainty equations. And, Sec. 5 presents the results of the MEMS Young’s modulus and step height round robin experiment followed by the conclusions in Sec. 6. Instrument specifications are given in Appendix A (Sec. 7). Supplemental material for Young’s modulus measurements can be found in Appendix B (Sec. 8), and supplemental material for step height measurements can be found in Appendix C (Sec. 9).

## 2. Packaged Round Robin Test Chip

The MEMS Round Robin Test Chip was fabricated on a 1.5 μm CMOS process available through MOSIS [20]. The design for this test chip is depicted in Fig. 1. The design file (in GDS-II format) for this chip can be downloaded from the NIST Semiconductor Electronics Division (SED) MEMS Calculator website [10].

For the round robin chip design shown in Fig. 1, in a number of places one mechanical layer is fabricated into suspended structures such as cantilevers and fixed-fixed beams. This layer consists of all oxide; namely, the field oxide, the deposited oxide before and after the first metal (m1) deposition, and the glass (gl) layer. [The nitride cap (present atop the glass layer when the chips are received from MOSIS) was removed after fabrication using a CF_4_ + O_2_ etch before a post-processing XeF_2_ etch that released the beams.]

As seen in Fig. 1, the test chip contains six groupings of test structures with the following labels:

1. Young’s Modulus,
2. Residual Strain,
3. Strain Gradient,
4. Step Height,
5. In-Plane Length, and
6. Certification Plus.

However, for the MEMS Young’s modulus and step height round robin experiment, we were only concerned with the first and fourth groupings of test structures, for Young’s modulus and step height measurements, respectively.

In the first grouping of test structures on the Round Robin Test Chip shown in Fig. 1, Young’s modulus measurements were made. Cantilever and fixed-fixed beam test structures were provided for this purpose with 25 cantilevers grouped above 25 fixed-fixed beams on this chip; however, for the round robin we were only concerned with the cantilevers, such as that shown in Fig. 2. Configurations for the cantilevers on the chip shown in Fig. 1 are given in Table 1. (See Sec. 3.1 for the rationale for the chosen cantilever dimensions.)

As shown in Table 1, the cantilever design lengths are 200 μm, 248 μm, 300 μm, 348 μm, and 400 μm. The length of the cantilever (in micrometers) is given at the top of each column of cantilevers in Fig. 1 following the column number (i.e., 1 to 5). The beams are designed at only a 0° orientation.^4^ There are five cantilevers designed at each length. Therefore, there are 25 oxide cantilevers with a 0° orientation.

In the fourth grouping of test structures on the Round Robin Test Chip shown in Fig. 1, step height measurements are made. Step height test structures, such as shown in Fig. 3, are provided for this purpose for a metal2-over-poly1 (m2-over-p1) step from active area (aa) to field oxide (fox). The surrounding reference platform consists of the deposited oxides and metal2 over active area. The metal2 thickness is approximately 1.0 μm.

**Fig. 4:**
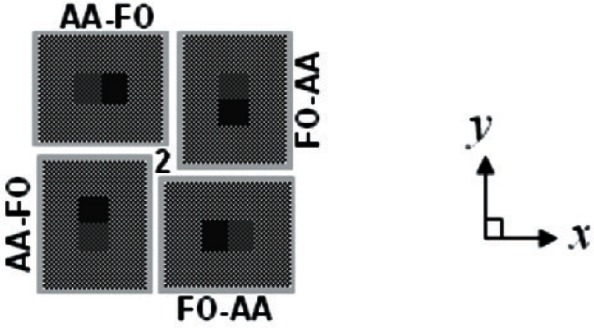
A design rendition of quad “2” from the step height grouping of test structures in Fig. 1.

There are four orientations (0°, 90°, 180°, and 270°) of the step height test structure shown in Fig. 3(a), and these orientations are grouped in quads. For the Round Robin Test Chip in Fig. 1 there are three quads, one of which is shown in Fig. 4. The quad number is given in the center of the quad. Therefore, quad “2” (out of 3) is shown in Fig. 4.

After the chips were received from MOSIS, they were post-fabricated in a class 100 clean room (to remove the nitride cap with a CF_4_ + O_2_ etch and to release the beams with a XeF_2_ etch), packaged in a laboratory environment, then stored in a dust-free N_2_ atmosphere.

**Fig. 5:**
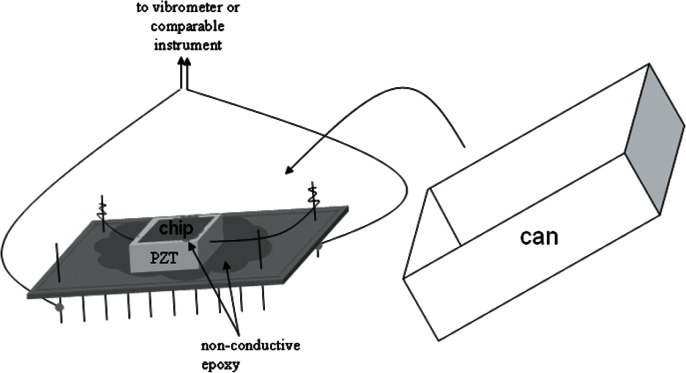
The packaged Round Robin Test Chip.

Each round robin participant received a packaged Round Robin Test Chip, as shown in Fig. 5. The packaged part was put together in the following way:

1. Starting with a hybrid package with a pin arrangement similar to that shown in Fig. 5, the PZT was secured to the top of the chip cavity using two thin layers of low stress, non-conducting epoxy. (The first layer of epoxy ensures that there will not be a conducting path between the package and the PZT.)
2. The PZT has the following properties:
  - The dimensions of the PZT are approximately 5 mm by 5 mm and 2 mm in height,
  - It is provided with a red and a black wire,
  - It can achieve a 2.2 μm (± 20 %) displacement at 100 V,
  - It has an electrical capacitance of 250 nF (± 20 %), and
  - It has a resonance frequency greater than 300 kHz, at which or above which it shall not be operated because it could damage the PZT.
3. The two PZT wires were secured to their respective package connections.
4. The Round Robin Test Chip was secured to the top of the PZT using two thin layers of a low stress non-conducting epoxy. (The first layer of epoxy ensures that there will not be a conducting path between the PZT and the test chip.)
5. The lid (or can) was placed on top of the package to protect the chip, and the can was secured to the package with tape before shipment.

To take measurements on the Round Robin Test Chip, the can is carefully removed. Young’s modulus and step height measurements can now be taken. For Young’s modulus measurements, to operate the PZT, the red wire is driven with a voltage that is positive relative to the black wire. To ensure that you have successfully connected to the PZT, when activated at 10 V and 7000 Hz, the resulting PZT vibration is barely audible.

## 3. Young’s Modulus and Step Height Measurements

The technical basis for the standard test method on Young’s modulus measurements is given in Sec. 3.1, and the technical basis for the standard test method on step height measurements is given in Sec. 3.2.

### 3. 1. Young’s Modulus Measurements

The Young’s modulus of a single layer is obtained from resonance frequency measurements of a cantilever comprised of that layer (such as shown in Fig. 2) or from resonance frequency measurements of a fixed-fixed beam comprised of that layer. To determine an estimate for the fundamental resonance frequency of a cantilever, *f*_caninit_, the following equation (as derived in Sec. 8.1) is used:^5^

(1) $$f_{caninit}=\sqrt{\frac{E_{init}t^{2}}{38.330\rho L_{can}^{4}}},$$

where *E_init_* is the initial estimate for the Young’s modulus value of the thin film layer, *t* is the thickness, *ρ* is the density, and *L_can_* is the suspended length of the cantilever.

Measurements are taken at frequencies which encompass *f_caninit_*, and an excitation-magnitude versus frequency plot is obtained from which the resonance frequency is found. For a given cantilever, three measurements of resonance frequency are obtained (namely, *f*_meas1_, *f*_meas2_, and *f*_meas3_). If these are undamped measurements (e.g., if they are performed in a vacuum) they are called *f*_undamped1_, *f*_undamped2_, and *f*_undamped3_, respectively. If these are damped measurements they are called *f*_damped1_, *f*_damped2_, and *f*_damped3_, respectively. For each damped frequency (*f*_damped1_, *f*_damped2_, and *f*_damped3_), a corresponding undamped frequency (*f*_undamped1_, *f*_undamped2_, and *f*_undamped3_, respectively) is calculated using the equation below:

(2) $$f_{\text{undamped}\;n}=\frac{f_{\text{damped}}{}_{n}}{\sqrt{1-\frac{1}{4Q^{2}}}},$$

where the *n* in the subscript of *f*_damped_*_n_* and *f*_undamped_*_n_* is 1, 2, or 3 and where *Q* is the oscillatory quality factor of the cantilever as given by the following equation [21]:

(3) $$Q=\left[\frac{W_{can}\sqrt{E_{init}\rho }}{24\mu }\right]{\left(\frac{t}{L_{can}}\right)}^{2},$$

where *W_can_* is the suspended cantilever width and *μ* is the viscosity (in air, *μ* = 1.84 × 10^–5^ Ns/m^2^ at 20 °C).

The cantilever dimensions for the Round Robin Test Chip in Fig. 1, as specified in Table 1, were chosen such that 5 μm ≤ *W_can_* ≤ 40 μm, *W_can_* > *t*, and *L_can_* ≫ *t* where *t* = 2.743 μm, as determined by the electro-physical technique [18]. Data Sheet T.1 [10] can be used to calculate *t*. In addition, the cantilever dimensions were chosen to achieve a) an estimated resonance frequency between 10 kHz and 75 kHz using Eq. (1) with the assumptions that *E_init_* = 70 GPa and *ρ* = 2.2 g/cm^3^, b) a *Q* value above 30 using Eq. (3), and c) a value less than 2 % for *p_diff_* as given by the following equation:

(4) $$p_{di\mathrm{ff}}=\left(1-\frac{f_{\text{damped}\;n}}{f_{\text{undamped}\;n}}\right)100\%=\left(1-\sqrt{1-\frac{1}{4Q^{2}}}\right)100\%.$$

See Table 2 for the calculations of *f_caninit_*, *Q*, and *p_diff_* for the chosen dimensions.

Also, to ensure that the resonance frequency of the cantilever is not altered by squeeze film or other damping phenomena,^6^ the cantilever should be suspended high enough above the underlying layer such that its motion is not altered by the underlying layer.^7^ In other words, the gap, *d*, between the suspended cantilever and the underlying layer should adhere to the following equation [22]:

(5) $$d\geq \frac{W_{can}}{3}.$$

Therefore, if *W_can_* = 28 μm, the gap between the suspended cantilever and the underlying layer should be at least 9.3 μm.

The average undamped resonance frequency, *f_can_*, is calculated from the three undamped resonance frequencies using the following equation:

(6) $$f_{can}=\frac{f_{\text{undamped}1}+f_{\text{undamped}2}+f_{\text{undamped}3}}{3}.$$

Given the measured value for *f_can_*, the Young’s modulus value, *E*, is calculated as follows:

(7) $$E=\frac{48\pi^{2}}{1.875^{4}}\frac{\rho f_{can}^{2}L_{can}^{4}}{t^{2}}=\frac{38.330\rho f_{can}^{2}L_{can}^{4}}{t^{2}},$$

which assumes clamped-free boundary conditions and no undercutting of the beam. The derivation of this equation is presented in Sec. 8.1. The combined standard uncertainty for *E*, or *u*_c_*_E_*, is given in Sec. 4.2. Residual stress and stress gradient equations for this thin film layer can be found in Sec. 8.2. Consult Sec. 8.3 for Young’s modulus measurements obtained from fixed-fixed beams.

### 3.2 Step Height Measurements

Step height measurements can be taken from either one or two step height test structures; however, measurements from one step height test structure are recommended due to lower resulting values for the combined standard uncertainty, *u*_cSH_ [17].

If one step height test structure is used to obtain a step height measurement, three 2-D data traces [“a,” “b,” and “c,” as shown in Fig. 3(a)] are taken along the top of the test structure, a cross section of which is given in Fig. 3(b). A sample 2-D data trace is shown in Fig. 3(c). All height measurements are with respect to the height of the surrounding reference platform that is used to level and zero the data. For generic test structure “*N*” with platforms labelled “*X*” and “*Y*,” the individual platform height measurements (namely, *platNX*a, *platNX*b, *platNX*c, *platNY*a, *platNY*b, and *platNY*c) and the standard deviations (*σ_platNX_*_a_, *σ_platNX_*_b_, *σ_platNX_*_c_, *σ_platNY_*_a_, *σ_platNY_*_b_, and *σ_platNY_*_c_) from the two platforms involved in the step in data traces “a,” “b,” and “c” are recorded.^8^ If the test structure in Fig. 3(a) is called test structure “1,” then for the step in test structure “1” from platform “A” to platform “B,” the platform height measurements would be *plat*1Aa, *plat*1Ab, *plat*1Ac, *plat*1Ba, *plat*1Bb, and *plat*1Bc, and the standard deviations would be *σ_plat_*_1Aa_, *σ_plat_*_1Ab_, *σ_plat_*_1Ac_, *σ_pla_*_t1Ba_, *σ_plat_*_1Bb_, and *σ_plat_*_1Bc_. Therefore, 12 measurements are obtained (6 from platform “A” and 6 from platform “B”).

For each 2-D data trace, the difference in the platform heights involved in a step (in general, *stepN_XYt_*)^9^ is calculated using the following equation:

(8) $$stepN_{XYt}=platNYt-platNXt$$

where *t* is the data trace (“a,” “b,” “c,” etc.) being examined. For the step shown in Fig. 3(b) from platform “A” to platform “B,” the equations are:

(9) $$step1_{\text{ABa}}=plat1\text{Ba}-plat1\text{Aa},$$

(10) $$step1_{\text{ABb}}=plat1\text{Bb}-plat1\text{Ab},$$

and

(11) $$step1_{\text{ABc}}=plat1\text{Bc}-plat1\text{Ac}.$$

The step height, *stepN_XY_*, is the average of the values from the different 2-D data traces as given below:

(12) $$stepN_{XY}=\frac{stepN_{XYa}+stepN_{XYb}+stepN_{XYc}}{3},$$

or for the step shown in Fig. 3(b), the step height, *step1*_AB_, is:

(13) $$step1_{\text{AB}}=\frac{step1_{\text{ABa}}+step1_{\text{ABb}}+step1_{\text{ABc}}}{3}.$$

The combined standard uncertainty, *u*_cSH_, for *stepN_XY_* is given in Sec. 4.3. Consult Sec. 9. 1 for a step height measurement obtained from two step height test structures.

## 4. Uncertainty Equations

In this section, the equations used to determine the values of the combined standard uncertainty [17], *u_c_*, are presented. Sec. 4.1 presents the basic combined standard uncertainty equation, and Sec. 4.2 and Sec 4.3 present the more specific uncertainty equations for Young’s modulus and step height measurements, respectively.

### 4.1 Combined Standard Uncertainty Equation

The combined standard uncertainty, *u_c_*, [17] is calculated as the estimated standard deviation of the result. It is equal to the square root of the sum of the squares of the uncertainty components where each component must have the same units as *u_c_* (e.g., GPa).

For the case of three sources of uncertainty, the uncertainty equation would be as follows:

(14) uc=u12+u22+u32

where *u*_1_ is the uncertainty component due to the first source of uncertainty, *u*_2_ is the uncertainty component due to the second source of uncertainty, and *u*_3_ is due to the third source of uncertainty. Additional terms may be added under the square root sign for additional sources of uncertainty.

### 4.2 Young’s Modulus Uncertainty Equations

In this section, a combined standard uncertainty equation is presented for use with Young’s modulus measurements (*u_cE_*) as obtained from resonance frequency measurements from a cantilever. For these measurements, six sources of uncertainty are identified with all other sources considered negligible. The six sources of uncertainty are the uncertainty of the thickness (*u_thick_*), the uncertainty of the density (*u_ρ_*), the uncertainty of the cantilever length (*u_L_*), the uncertainty of the average resonance frequency (*u_freq_*), the uncertainty due to the frequency resolution (*u_fresol_*), and the uncertainty due to damping (*u_damp_*). As such, the combined standard uncertainty equation for *u_cE_* (as calculated in SEMI Test Method MS4 [5] and in Data Analysis Sheet YM. 1 [10]) with six sources of uncertainty is as follows:

(15) $$u_{cE}=\sqrt{u_{thick}^{2}+u_{\rho }^{2}+u_{L}^{2}+u_{freq}^{2}+u_{fresol}^{2}+u_{damp}^{2}}.$$

In determining the combined standard uncertainty, a Type B evaluation [17] (i.e., one that uses means other than the statistical Type A analysis) is used for each source of error. Table 3 gives sample values for each of these uncertainty components.

The uncertainty for *u_thick_* is determined from calculated minimum and maximum Young’s modulus values (namely, *E*_min_ and *E*_max_, respectively). It is derived using the extremes of values expected for the cantilever thickness as given below:

(16) $$E_{\min}=\frac{38.330\rho f_{can}^{2}L_{can}^{4}}{{\left(t+3\sigma_{thick}\right)}^{2}}$$

and

(17) $$E_{\max}=\frac{38.330\rho f_{can}^{2}L_{can}^{4}}{{\left(t-3\sigma_{thick}\right)}^{2}},$$

where *σ_thick_* is the one sigma uncertainty of the value of *t*, which is found using the electro-physical technique [18]. Data Sheet T.1 [10] can be used to calculate *σ_thick_*. With 99.7 % confidence, assuming a Gaussian distribution (and assuming *u_ρ_*, *u_L_*, *u_freq_*, *u_fresol_*, and *u_damp_* equal zero), the value for *E* lies between *E*_min_ and *E*_max_. Therefore, *u_thick_* is calculated as follows:

(18) $$u_{thick}=\frac{E_{\max}-E_{\min}}{6}.$$

In the same way, to determine the uncertainty component, *u_ρ_*, the calculated minimum and maximum Young’s modulus values (namely, *E*_min_ and *E*_max_, respectively) are given below:

(19) $$E_{\min}=\frac{38.330\;(\rho -3\sigma_{\rho })f_{can}^{2}L_{can}^{4}}{t^{2}}$$

and

(20) $$E_{\max}=\frac{38.330\;(\rho +3\sigma_{\rho })f_{can}^{2}L_{can}^{4}}{t^{2}},$$

where *σ_ρ_* is the estimated one sigma uncertainty of the value of *ρ*. The uncertainty component, *u_ρ_*, is therefore calculated in the same way as before:

(21) $$u_{\rho }=\frac{E_{\max}-E_{\min}}{6}.$$

The uncertainty equation for *u_L_* is determined from the minimum and maximum Young’s modulus values (namely, *E*_min_ and *E*_max_, respectively) as given below:

(22) $$E_{\min}=\frac{38.330\rho f_{can}^{2}{\left(L_{can}-3\sigma_{L}\right)}^{4}}{t^{2}}$$

and

(23) $$E_{\max}=\frac{38.330\rho f_{can}^{2}{\left(L_{can}+3\sigma_{L}\right)}^{4}}{t^{2}},$$

where *σ_L_* is the estimated one sigma uncertainty of the value of *L_can_*. Therefore, *u_L_* is calculated as follows:

(24) $$u_{L}=\frac{E_{\max}-E_{\min}}{6}.$$

The uncertainty equation for *u_freq_* is determined from the minimum and maximum Young’s modulus values (namely, *E*_min_ and *E*_max_, respectively) as given below:

(25) $$E_{\min}=\frac{38.330\rho {\left(f_{can}-3\sigma_{freq}\right)}^{2}L_{can}^{4}}{t^{2}}$$

and

(26) $$E_{\max}=\frac{38.330\rho {\left(f_{can}+3\sigma_{freq}\right)}^{2}L_{can}^{4}}{t^{2}},$$

where *σ_freq_* is the one sigma uncertainty of the value of *f_can_*. Therefore, *u_freq_* is calculated as follows:

(27) $$u_{freq}=\frac{E_{\max}-E_{\min}}{6}.$$

The uncertainty equation for *u_fresol_* is determined from the minimum and maximum Young’s modulus values (namely, *E*_min_ and *E*_max_, respectively) as given below:

(28) $$E_{\min}=\frac{38.330\rho {\left(f_{can}-\frac{f_{resol}}{2}\right)}^{2}L_{can}^{4}}{t^{2}}$$

and

(29) $$E_{\max}=\frac{38.330\rho {\left(f_{can}+\frac{f_{resol}}{2}\right)}^{2}L_{can}^{4}}{t^{2}},$$

where *f_resol_* is the frequency resolution for the given set of measurement conditions. Assuming a uniform (i.e., rectangular) probability distribution (and assuming *u_thick_*, *u_ρ_*, *u_L_*, *u_freq_*, and *u_damp_* equal zero), the value for *E* lies between *E*_min_ and *E*_max_. Therefore, *u_fresol_* is calculated as follows:

(30) $$u_{fresol}=\frac{E_{\max}-E_{\min}}{2\sqrt{3}}.$$

If undamped resonance frequencies (e.g., if the measurements were performed in a vacuum) were recorded as *f*_meas1_, *f*_meas2_, and *f*_meas3_, then *u_damp_* is set equal to 0.0 Pa. For damped resonance frequencies (i.e., if *f*_meas1_, *f*_meas2_, and *f*_meas3_ were damped measurements), the uncertainty equation for *u_damp_* is determined from the minimum and maximum Young’s modulus values (namely, *E*_min_ and *E*_max_, respectively) as given below:

(31) $$E_{\min}=\frac{38.330\rho {\left(f_{can}-3\sigma_{fQ}\right)}^{2}L_{can}^{4}}{t^{2}}$$

and

(32) $$E_{\max}=\frac{38.330\rho {\left(f_{can}+3\sigma_{fQ}\right)}^{2}L_{can}^{4}}{t^{2}},$$

where the calculation for *σ_fQ_* is given in Sec. 8.6. Therefore, *u_damp_* is calculated as follows:

(33) $$u_{damp}=\frac{E_{\max}-E_{\min}}{6}.$$

### 4.3. Step Height Uncertainty Equations

In this section, a combined standard uncertainty equation is presented for use with step height measurements (*u*_cSH_). Six sources of uncertainty are identified with all other sources of uncertainty considered negligible. The six sources of uncertainty are the uncertainty of the measurement across the length of the step (*u_L_*_step_) where the length is measured perpendicular to the edge of the step, the uncertainty of the measurement across the width of the step (*u_W_*_step_) where the width is measured parallel to the edge of the step, the uncertainty of the value of the physical step height used for calibration (*u_cert_*), the uncertainty of the measurement due to the repeatability (*u*_repeat_), the uncertainty due to the amount of drift during the data session (*u*_drift_), and the uncertainty of a measurement due to the deviation from height linearity of the data scan (*u*_linear_). As such, the combined standard uncertainty equation (as calculated in SEMI Test Method MS2 [6] and in Data Analysis Sheet SH.1 [10]) with six sources of uncertainty is as follows:

(34) $$u_{\text{cSH}}=\sqrt{u_{L\text{step}}^{2}+u_{W\text{step}}^{2}+u_{cert}^{2}+u_{\text{repeat}}^{2}+u_{\text{drif}\;t}^{2}+u_{\text{linear}}^{2}}.$$

In determining the combined standard uncertainty, a Type B evaluation [17] (i.e., one that uses means other than the statistical Type A analysis) is used for each Type B source of error, except where noted. Table 4 gives sample values for each of these uncertainty components.

The uncertainty equation for *u_L_*_step_ assuming a Gaussian distribution is as follows:

(35) $$u_{Lstep}=\sqrt{{\left(\sigma_{platNX\text{ave}}-\sigma_{\text{rough}NX}\right)}^{2}+{\left(\sigma_{platNY\text{ave}}-\sigma_{\text{rough}NY}\right)}^{2}},$$

where *σ*_rough_*_NX_* and *σ*_rough_*_NY_* are the surface roughnesses of *platNX* and *platNY*, respectively, and are calculated from the smallest of all the calibrated values obtained for *σ_platNXt_* and *σ_platNYt_*, respectively. However, if the surfaces of *platNX* (defined in the List of Symbols), *platNY*, and *platN*r all have identical compositions, then *σ*_rough_*_NX_* equals *σ*_rough_*_NY_*, which equals the smallest of all the values obtained for *σ_platNXt_*, *σ_platNYt_*, and *σ_platN_*_r_*_Dt_*.^10^ Also, in the above equation, *σ_platNX_*_ave_ and *σ_platNY_*_ave_ are calculated using the following equations:

(36) $$\sigma_{platNX\text{ave}}=\frac{\sigma_{platNXa}+\sigma_{platNXb}+\sigma_{platNXc}}{3}$$

and

(37) $$\sigma_{platNY\text{ave}}=\frac{\sigma_{platNYa}+\sigma_{platNYb}+\sigma_{platNYc}}{3}.$$

The derivation of *u_Lstep_* is given in Sec. 9.3.

The uncertainty equation for *u_Wstep_* is determined from *σ_Wstep_*, the one sigma standard deviation of the step height measurements *stepN_XY_*_a_, *stepN_XY_*_b_, and *stepN_XY_*_c_, using the following equation:

(38) $$u_{Wstep}=\sigma_{Wstep}.$$

This is a statistical Type A component.

The uncertainty equation for *u_cert_* is determined from the minimum and maximum step height values (namely, *stepN_XY_*_min_ and *stepN_XY_*_max_, respectively). The uncertainty of the measured step height is assumed to scale linearly with height. With 99.7 % confidence, assuming a Gaussian distribution (and assuming *u_Lstep_*, *u_Wstep_*, *u*_repeat_, *u*_drift_, and *u*_linear_ equal zero), the value for |*stepN_XY_*| lies between *stepN_XY_*_min_ and *stepN_XY_*_max_. As such, *u_cert_* can be calculated using the following equation:

(39) $$u_{cert}=\frac{\sigma_{cert}}{cert}|stepN_{XY}|$$

where *cert* is the certified value of the double-sided physical step height used for calibration and *σ_cert_* is the one sigma uncertainty of the calibrated physical step height.

The uncertainty equation for *u*_repeat_ is determined from the minimum and maximum step height values (namely, *stepN_XY_*_min_ and *stepN_XY_*_max_, respectively) as given below:

(40) $$stepN_{XY\min}=|stepN_{XY}|-|stepN_{XY}|\frac{z_{\text{repeat}}}{2{\bar{z}}_{6}}$$

and

(41) $$stepN_{XY\max}=|stepN_{XY}|+|stepN_{XY}|\frac{z_{\text{repeat}}}{2{\bar{z}}_{6}},$$

where *z*_repeat_ is the maximum of two calibrated values; one of which is the positive calibrated difference between the minimum and maximum values of the six calibration measurements taken at a single location on the calibration step before the data session and the other of which is the positive calibrated difference between the minimum and maximum values of the six calibration measurements taken at the same location on the calibration step after the data session and where ${\bar{z}}_{6}$ is the calibrated average of the six calibration measurements from which *z*_repeat_ is found. The uncertainty of the measured step height is assumed to scale linearly with height. Assuming a uniform distribution (and assuming *u_Lstep_*, *u_Wstep_*, *u_cert_*, *u*_drift_, and *u*_linear_ equal zero), the value for |*stepN_XY_*| lies between *stepN_XY_*_min_ and *stepN_XY_*_max_. Therefore, *u*_repeat_ is calculated as follows:

(42) $$u_{repeat}=\frac{stepN_{XY\max}-stepN_{XY\min}}{2\sqrt{3}},$$

which simplifies to the following equation:

(43) $$u_{repeat}=\frac{z_{repeat}}{2\sqrt{3}{\bar{z}}_{6}}|stepN_{XY}|.$$

The uncertainty equation for *u*_drift_ is determined from the minimum and maximum step height values (namely, *stepN_XY_*_min_ and *stepN_XY_*_max_, respectively) as given below:

(44) $$stepN_{XY\min}=|stepN_{XY}|-|stepN_{XY}|\frac{z_{\text{drift}}}{2\bar{z}}$$

and

(45) $$stepN_{XY\max}=|stepN_{XY}|+|stepN_{XY}|\frac{z_{\text{drift}}}{2\bar{z}},$$

where *z*_drift_ is the calibrated positive difference between the averages of the 6 calibration measurements taken before and after the data session (at the same location on the physical step height used for calibration) and where $\bar{z}$ is the calibrated average of all 12 calibration measurements. The uncertainty of the measured step height is assumed to scale linearly with height. Assuming a uniform distribution (and assuming *u_Lstep_*, *u_Wstep_*, *u_cert_*, *u*_repeat_, and *u*_linear_ equal zero), the value for |*stepN_XY_*| lies between *stepN_XY_*_min_ and *stepN_XY_*_max_. Therefore, *u*_drift_ is calculated as follows:

(46) $$u_{drift}=\frac{stepN_{XY\max}-stepN_{XY\min}}{2\sqrt{3}},$$

which simplifies to the following equation:

(47) $$u_{drift}=\frac{z_{drift}}{2\sqrt{3}\bar{z}}|stepN_{XY}|.$$

The uncertainty equation for *u*_linear_ is calculated from the minimum and maximum step height values (namely, *stepN_XY_*_min_ and *stepN_XY_*_max_, respectively) as given:

(48) $$stepN_{XY\min}=|stepN_{XY}|-|stepN_{XY}|z_{perc}$$

and

(49) $$stepN_{XY\max}=|stepN_{XY}|+|stepN_{XY}|z_{perc},$$

where *z*_perc_ is the maximum percent deviation from linearity over the instrument’s total scan range, as quoted by the instrument manufacturer. The uncertainty of the measured step height is assumed to scale linearly with height. Assuming a uniform distribution, *u_linear_* can be calculated using the following equation:

(50) $$u_{linear}=\frac{z_{perc}}{\sqrt{3}}|stepN_{XY}|.$$

Consult Sec. 9.2 for the uncertainty calculations when two step height test structures are used for a step height measurement.

## 5. Round Robin Results

The round robin repeatability and reproducibility results are given in Sec. 5.1 for Young’s modulus measurements and in Sec. 5.2 for step height measurements.

The repeatability measurements are performed using the same test method, in the same laboratory (NIST), by the same operator, with the same equipment, in the shortest practicable period of time. These measurements are done on random test structures.

For the reproducibility measurements, at least six independent data sets (each using a different piece of equipment or equipment setup) must be obtained following the same test method before the results can be recorded in the precision and bias statement of a SEMI standard test method. These measurements are done on random test structures, as described below.

### 5.1 Young’s Modulus Round Robin Results

For the Young’s modulus portion of the MEMS Young’s modulus and step height round robin experiment, both repeatability and reproducibility data were taken.

The repeatability data were taken at one laboratory using a dual beam vibrometer (see Sec. 7.1 for specifics associated with the vibrometer). Young’s modulus values were found from 12 different cantilevers 4 times, with each Young’s modulus value determined from the average of 3 resonance frequency measurements. Therefore, 48 Young’s modulus values were obtained. Of these values, 16 were from 4 different cantilevers with *L* = 200 μm, 16 from 4 different cantilevers with *L* = 300 μm, and 16 from 4 different cantilevers with *L* = 400 μm.

For the reproducibility data, 8 participants were identified.^11^ Each participant was supplied with a Round Robin Test Chip and asked to obtain a Young’s modulus value from 3 oxide cantilevers with design lengths of 200 μm, 300 μm, and 400 μm. (The participant could choose to measure any one of five cantilevers of the given length that were available on the test chip as long as it passed a visual inspection.) Each Young’s modulus value was determined from the average of three resonance frequency measurements from the cantilever as specified in Sec. 3.1, using an instrument that meets the manufacturer’s alignment and calibration criteria. Following SEMI MS4 for Young’s modulus measurements, the measurements were recorded on Data Analysis Sheet YM.1 [10].

The eight participants used a variety of instruments (consult Sec. 7.1 for details associated with the instruments) to obtain Young’s modulus. These included a single beam vibrometer, a dual beam vibrometer, and a stroboscopic interferometer. In addition, thermal excitation measurements are included for comparison with PZT excitation measurements on the same chip.

The repeatability and reproducibility data for Young’s modulus and for the combined standard uncertainty is presented in Table 5 and Table 6, respectively, where *n* indicates the number of calculated Young’s modulus values. The average of the repeatability or reproducibility data (namely *E*_ave_) is listed next, followed by the 95 % limits for *E* that is calculated as follows: (a) the standard deviations were found, (b) these values were multiplied by 2.0 (assuming a Gaussian distribution) [17], and (c) the resulting values were reported as percents. Below this, the average of the combined standard uncertainty [17] values (*u*_cave_) and the 95 % limits for *u*_c_*_E_* are presented.

**Fig. 6:**
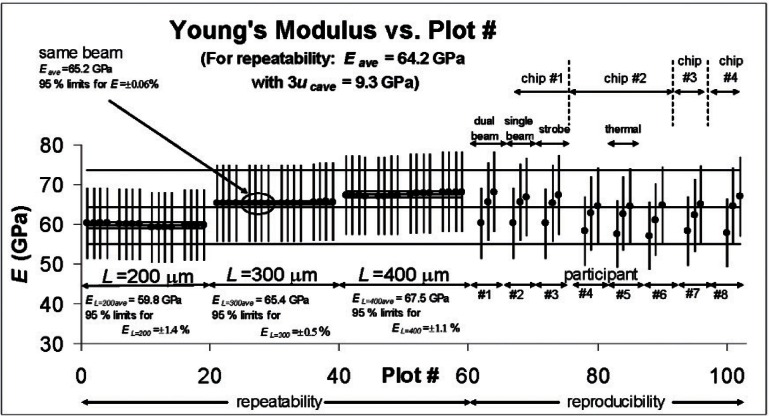
Young’s modulus round robin results.

The Young’s modulus round robin results are plotted in Fig. 6, where both the repeatability and reproducibility data are plotted. The average Young’s modulus value for the repeatability data is specified at the top of Fig. 6 along with the average 3*u*_c_*_E_* uncertainty bars for this value.^12^ These quantities are plotted in this figure with both the repeatability and reproducibility data. As an observation, all of the reproducibility results fall comfortably between the repeatability bounds of *E_ave_* plus or minus 3*u_cave_*.

In Fig. 6, the repeatability data are grouped according to the cantilever length with the *L* = 200 μm data plotted first, followed by the *L* = 300 μm data, then the *L* = 400 μm data. In like manner with the reproducibility data, for each participant, the *L* = 200 μm data are plotted first, followed by the *L* = 300 μm data, then the *L* = 400 μm data. The repeatability data and the reproducibility data both indicate a length dependency. The repeatability data in Fig. 6 show a clustering of the data at each length. In other words, the 95 % limits for *E* at each length (which are plotted in this figure along with *E_ave_* for each length) are all less than 1.5 %, which is much less than the 10.3 % value (as given in Table 5) when all the lengths are considered. This suggests that when Young’s modulus values extracted by different measurement instruments or excitation methods are compared, the cantilevers should have the same length. This length dependency can be due to a number of things including debris in the attachment corners of the cantilevers to the beam support, which would cause larger errors for shorter length cantilevers. This can be a topic for future investigation where a) the physical form and chemical composition of the cantilever is checked to see if it matches the assumptions used in the calculations and b) finite element methods are used to determine if the length dependency is due to the attachment conditions. Therefore, at this point, we can only state that, given the existing cantilevers, we can only report an “effective” value for Young’s modulus.

Round robin participant #1, participant #2, and participant #3 took measurements on the same chip (chip #1) using a dual beam vibrometer, a single beam vibrometer, and a stroboscopic interferometer, respectively. The results given in Fig. 6 indicate that comparable results were obtained from these instruments.

Round robin participant #4, participant #5, and participant #6 took data from the same chip (chip #2); however, round robin participant #5 used thermal excitation to obtain the required data while participant #4 and participant #6 used PZT excitation. No significant difference in the results for these measurements is seen in Fig. 6.

No information can be presented on the bias of the procedure in the test method for measuring Young’s modulus because there is not a certified MEMS material for this purpose. Many values for Young’s modulus for various materials have been published with an attempt to consolidate this information in [23]. For a silicon dioxide film, the Young’s modulus values reported in [23] range from 46 GPa to 92 GPa. The average repeatability value reported in Table 5 of 64.2 GPa falls comfortably within this range.

The Young’s modulus results are reported as follows [17]: Since it can be assumed that the possible estimated values are either approximately uniformly distributed or Gaussian (as specified in Sec. 4.2) with approximate standard deviation *u*_c_*_E_*, the Young’s modulus value is believed to lie in the interval *E* ± *u*_c_*_E_* with a level of confidence of approximately 68 % assuming a Gaussian distribution.

### 5.2 Step Height Round Robin Results

For the step height portion of the MEMS Young’s modulus and step height round robin experiment, both repeatability and reproducibility data were taken.

The repeatability data were taken at one laboratory using a stroboscopic interferometer operated in the static mode (see Appendix A). Four step height measurements were taken from each of the four test structures in each of the three quads shown in Fig. 1. Therefore, 48 step height measurements were obtained with 1 step height measurement defined as the average of 3 measurements taken from different positions somewhat evenly spaced along the step as specified in Sec. 3.2.

For the reproducibility data, seven participants were identified. Each participant was supplied with a Round Robin Test Chip and asked to obtain the step height from any two test structures in the first of the three quads of step height test structures. Following SEMI MS2 [6] for step height measurements, the raw, uncalibrated measurements were recorded on Data Analysis Sheet SH.1 [10].

The repeatability and reproducibility data for step height and for the combined standard uncertainty is presented in Table 7, where *n* indicates the number of step height measurements. The average of the repeatability or reproducibility data (namely |*stepN*_ABave_|) is listed next followed by the 95 % limits for |*stepN*_AB_| that is calculated as follows: (a) the standard deviations were found, (b) these values were multiplied by 2.0 (assuming a Gaussian distribution) [17], and (c) the resulting values were reported as percents. Below this, the average of the combined standard uncertainty [17] values (*u_cave_*) and the 95 % limits for *u_cSH_* are presented. (It is interesting in comparing the 95 % limits for |*stepN*_AB_| that the repeatability limits are larger than the reproducibility limits. The reason for this anomaly is not known.)

**Fig. 7:**
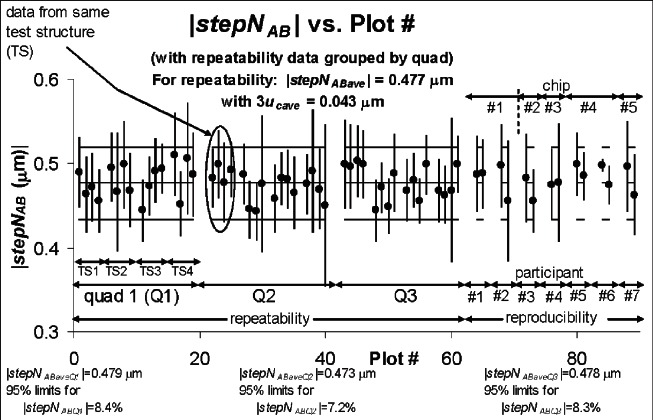
Step height round robin results with the repeatability results grouped according to quad

**Fig. 8:**
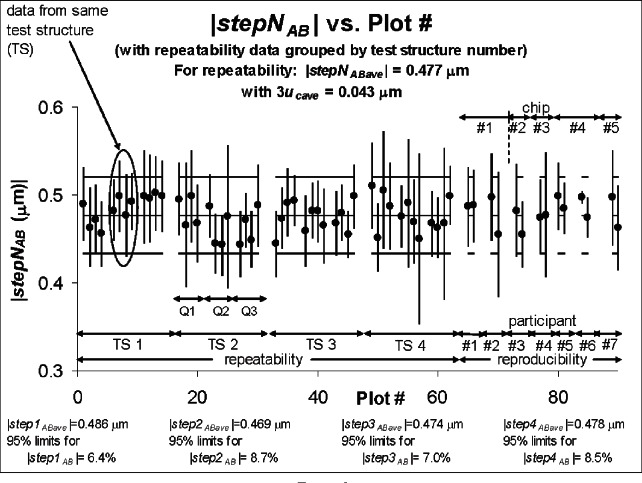
Step height round robin results with the repeatability results grouped according to test structure number.

The step height round robin results are plotted in Fig. 7 and Fig. 8. In each of these figures, the repeatability data are plotted first, followed by the results from the seven participants.^13^ The absolute value of the average step height for the repeatability data is specified at the top of Fig. 7 and Fig. 8 along with the 3*u_cave_* uncertainty bars for this value, as obtained or derived from Table 7.^14^ These quantities are plotted in each figure with both the repeatability and reproducibility data. As an observation, all of the reproducibility results fall comfortably between |*stepN*_ABave_| plus or minus 3*u_cave_* as obtained from the repeatability results.

Figure 7 groups the repeatability results by quad number with the results from quad “1” plotted first, followed by the results from quad “2,” then the results from quad “3.” The results within each quad are grouped according to test structure number^15^ with the results from test structure “1” plotted first, followed by the results from test structure “2,” etc. The average step height value and the 95 % limits for this value for each quad are given at the bottom of Fig. 7 and also in Table 8. These results reveal comparable values for the step height measurements and comparable values for the 95 % limits. This implies there are no discernable variations in the step height value between neighboring quads.

Figure 8 groups the repeatability results by test structure number with the results from test structure “1” (TS1) plotted first, followed by the results from test structure “2” (TS2), followed by the results from test structure “3” (TS3), then test structure “4” (TS4). The results for each test structure number are grouped according to quad with the results from quad “1” plotted first, followed by the results from quad “2,” then the results from quad “3.” As in Fig. 7, the average step height value and the 95 % limits for this value for each test structure are given at the bottom of Fig. 8 and also in Table 9. These results reveal that TS1 and TS3 (which are rotated ± 90° with respect to TS2 and TS4) have comparable 95 % limits as do TS2 and TS4; however the 95 % limits for TS1 and TS3 are slightly less than the 95 % limits for TS2 and TS4 when they should be comparable. There are also more variations in the average step height value between rotated test structures (as shown in Fig. 8 and Table 9) than variations in this value between quads (as shown in Fig. 7 and Table 8).

The platform surfaces involved in the step were not ideal surfaces. Oftentimes they were tilted (even though the data were leveled with respect to the reference platform) and the data jagged. Therefore, the precise selection of the analysis regions (including the number of data points within these regions) affects the standard deviations obtained. An averaging capability incorporated in most non-contact instruments can have the effect of smoothing the data; however, a more comprehensive determination of the length and width variations may be necessary when dealing with tilt. Repeatability might also be improved by calculating the step height from fitted straight lines as described for NIST step height calibrations [24] and outlined in ASTM E2530 [25]. As given in [24], “For step height measurements, one of several algorithms may be used. For single-sided steps, a straight line is fitted by the method of least squares to each side of the step transition, and the height is calculated from the relative position of these two lines extrapolated to the step edge.”

The interferometer or comparable instrument is calibrated in the out-of-plane *z*-direction. If the calibration is not done, a bias to the measurements is expected. The direction and degree of the resulting bias is different for each magnification of each instrument. As such, calibration of the interferometer or comparable instrument is considered mandatory for step height measurements.

The step height results are reported as follows [17]: Since it can be assumed that the possible estimated values are either approximately uniformly distributed or Gaussian (as specified in Sec. 4.3) with approximate standard deviation *u_cSH_*, the step height is believed to lie in the interval *stepN_XY_* ± *u_cSH_* with a level of confidence of approximately 68 % assuming a Gaussian distribution.

## 6. Conclusions

The technical basis for the two SEMI standard test methods on Young’s modulus measurements [5] and step height measurements [6] was presented, along with the data obtained from the MEMS Young’s modulus and step height round robin experiment. These data were incorporated into the precision and bias statements for the applicable SEMI standards.

For Young’s modulus measurements, data obtained from a single beam vibrometer, a dual beam vibrometer, and a stroboscopic interferometer yielded comparable results. Also, PZT excitation and thermal excitation yielded comparable results. The repeatability data and the reproducibility data both indicate a length dependency. In other words, the 95 % limits for *E* at each length are all less than ±1.5 %, which is much less than the ±10.3 % value when all the lengths from 200 μm to 400 μm are considered. This could be due to debris in the attachment corners of the cantilevers to the beam support that would cause larger errors for shorter length cantilevers. Additional research can ascertain if the physical form and chemical composition of the cantilever matches the assumptions used in the calculations and if the length dependency is due to the attachment conditions. Therefore, at this point, we can only state that, given the existing cantilevers, we can only report an “effective” value for Young’s modulus. Also, when Young’s modulus values extracted by different measurement instruments or excitation methods are compared, the cantilevers should have the same length.

For step height measurements, it is currently not understood why the repeatability limits (± 7.9 %) are larger than the reproducibility limits (± 6.2 %). The platform surfaces involved in the step are not ideal surfaces, such that the precise selection of the analysis regions (including the number of data points within these regions) affects the standard deviations obtained. Also, the repeatability data indicate there are no discernable variations in the step height value between neighboring quads. And, there are more variations in the average step height value between rotated test structures than variations in this value between quads. For one-sided steps, the large uncertainties can be improved by calculating the step height from fitted straight lines (using the method of least squares) with the height calculated from the relative position of the two lines extrapolated to the step edge. This approach is typically used for step height measurements and calibrations [24,25].

It is expected that these standards will be instrumental in reducing the interlaboratory differences in the parametric measurements. The following guidelines should be of value to the MEMS industry, when communicating data obtained using these SEMI standard test methods:

- To record the data and perform the calculations, use the data analysis sheets which are accessible via the NIST SED MEMS Calculator website [10],
- Make sure the data have passed the verification check given at the bottom of the data analysis sheets, and
- Ask if all the verification checks were passed, when communicating with others concerning SEMI standard data.

Consult Ref. [10] for information associated with the MNT 5-in-1 SRM that enables companies to compare their in-house measurements taken on this SRM with NIST measurements thereby validating their use of the documentary standards.

## Figures and Tables

**Fig. 9:**
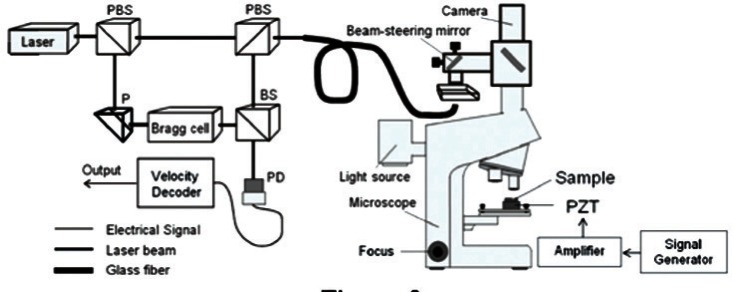
Schematic of a typical setup for a single beam laser vibrometer. (PBS indicates a polarizing beam splitter, BS indicates a beam splitter, P indicates a prism, and PD indicates a photodetector.)

**Fig. 10:**
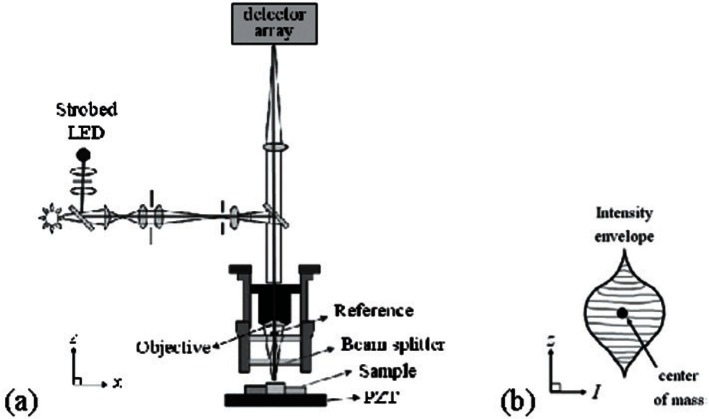
For a typical stroboscopic interferometer (a) a schematic and (b) an intensity envelope used to obtain a pixel’s sample height.

**Fig. 11:**
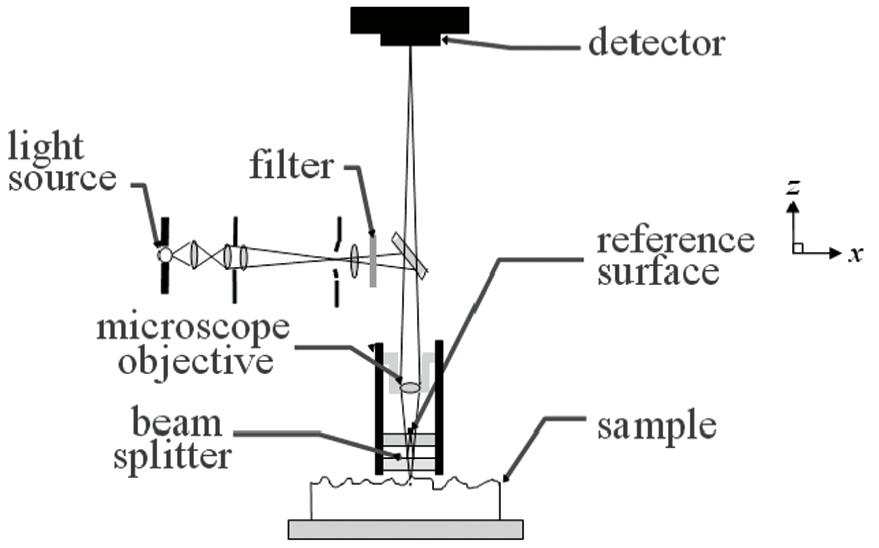
Schematic of an optical interferometric microscope operating in the Mirau configuration where the beam splitter and the reference surface are between the microscope objective and the sample.

**Table 1:** Cantilever configurations for Young’s modulus measurements

Test Structure	Cantilevers
Width (in μm)	28
Length (in μm)	200, 248, 300, 348, and 400
Orientation	0°
Mechanical Layer	Oxide
Quantity of Beams	5 of each length (or 25 beams)

**Table 2:** Calculations of *f*_caninit_, *Q*, and *p_diff_* for the designed cantilever lengths (*W*_can_ = 28 μm)

*L*_can_ (μm)	*f_caninit_* (kHz)	*Q*	*p_diff_* (%)
200	62.5	148.0	0.0006
248^a^	40.6	96.3	0.0013
300	27.8	65.8	0.0029
348^a^	20.6	48.9	0.0052
400	15.6	37.0	0.0091

a These values were chosen in order to design on a 0.8 μm grid to simplify the interface with MOSIS and the fabrication facility.

**Table 3:** Sample Young’s modulus uncertainty values (assuming *E_init_* = 70 GPa)

uncertainty		Type A or Type B	value (in GPa)
*u*thick	(using *t* = 2.743 μm and *σ*_thick_ = 0.058 μm)	Type B	2.8
*u_ρ_*	(using *ρ* = 2.2 g / cm^3^ and *σ_ρ_* = 0.05 g/cm^3^)	Type B	1.5
*u_L_*	(using *L*_can_ = 300 μm and *σ_L_* = 0.2 μm)	Type B	0.17
*u*_freq_	(using *f*_meas1_ = 26.82625 kHz, *f*_meas2_ = 26.8351 kHz, and *f*_meas3_ = 26.8251 kHz)	Type B	0.027
*u*_fresol_	(using *f*_resol_ = 1.25 Hz)	Type B	0.0018
*u*_damp_	(using *W_can_* = 28 μm and *σ_W_* = 0.1 μm and using *μ* = 1.84 × 10^−5^ Ns / m^2^ and *σ_μ_* = 0.01 × 10^−5^ Ns / m^2^)	Type B	0.0004
*u*_c_*_E_*	$=\sqrt{u_{\text{thick}}^{2}+u_{\rho }^{2}+u_{L}^{2}+u_{\text{freq}}^{2}+u_{\text{fresol}}^{2}+u_{\text{damp}}^{2}}$		3.2
$3u_{cE}{}^{a}$			9.5

a This 3*u*_c_*_E_* uncertainty is plotted in Fig. 6 with the repeatability data point corresponding to the first cantilever with length of 300 μm.

**Table 4:** Sample step height uncertainty values

uncertainty		Type A or Type B	value (in μm)
*u_Lstep_*	(using *σ_platNX_*_ave_ = 0.0118 μm, *σ_platNY_*_ave_ = 0.0102 μm, and *σ*_rough_*_NX_* =*σ*_rough_*_NY_* = 0.0036 μm)	Type B	0.011
*u_Wstep_*	(using *stepN_XY_*_a_ = 0.4928 μm, *stepN_XY_*_b_ = 0.4814 μm, and *stepN_XY_*_c_ = 0.4949 μm)	Type A	0.0073
*u_cert_*	(using *cert* = 9.887 μm, *σ_cert_* = 0.083 μm, and *stepN_XY_* = 0.490 μm)	Type B	0.0041
*u*_repeat_	(using *z*_repeat_ = 0.024 μm and ${\bar{Z}}_{6}=9.879\;\mu m$)	Type B	0.00034
*u*_drift_	(using *z*drift = 0.016 μm and $\bar{Z}=9.887\;\mu m$)	Type B	0.00023
*u*_linear_	(using *z*_perc_ = 1.0 %)	Type B	0.0028
*u*_cSH_	$=\sqrt{u_{Lstep}^{2}+u_{Wstep}^{2}+u_{cert}^{2}+u_{\text{repeat}}^{2}+u_{\text{drift}}^{2}+u_{\text{linear}}^{2}}$		0.014
$3u_{\text{cSH}}{}^{a}$			0.041

a This 3*u*_cSH_ uncertainty is associated with the first repeatability data point plotted in Fig. 7 (for TS1 in quad 1).

**Table 5:** Young’s modulus repeatability data (1 participant, 1 laboratory, 1 instrument, 1 chip, 12 different cantilevers)

	200 μm length	300 μm length	400 μm length	200 μm to 400 μm lengths
*n*	16	16	16	48
*E*_ave_ (in GPa)	59.8	65.4	67.5	64.2
95 % limits for *E*	± 1.4 %	± 0.5 %	± 1.1 %	± 10.3 %
*u*_cave_ (in GPa)	2.9 (4.9 %)	3.2 (4.8 %)	3.3 (4.8 %)	3.1 (4.8 %)
95 % limits for *u*_c_*_E_*	± 1.4 %	± 0.5 %	± 1.1 %	± 10.1 %

**Table 6:** Young’s modulus reproducibility data (eight participants, five laboratories, seven instruments, four chips)

	200 μm length	300 μm length	400 μm length	200 μm to 400 μm lengths
*n*	8	8	8	24
*E*_ave_ (in GPa)	58.7	63.7	66.0	62.8
95 % limits for *E*	± 4.4 %	± 5.5 %	± 4.4 %	± 11.0 %
*u*_cave_ (in GPa)	2.8 (4.9 %)	3.1 (4.8 %)	3.2 (4.9 %)	3.0 (4.9 %)
95 % limits for *u*_c_*_E_*	± 4.4 %	± 5.5 %	± 4.5 %	± 11.0 %

**Table 7:** Step height measurement results

	Repeatability results	Reproducibility results
*n*	48	14
|*stepN*_ABave_| (in μm)	0.477	0.481
95 % limits for |*stepN*_AB_|	± 7.9 %	± 6.2 %
*u*_cave_ (in μm)	0.014 (3.0 %)	0.014 (3.0 %)
95 % limits for *u*_cSH_	± 70 %	± 80 %

**Table 8:** Step height repeatability data grouped by quad

	Q1	Q2	Q3
*n*	16	16	16
|*stepN*_ABave_| (in μm)	0.479	0.473	0.478
95 % limits for |*stepN*_AB_|	± 8.4 %	± 7.2 %	± 8.3 %
*u*_cave_ (in μm)	0.015 (3.1 %)	0.015 (3.2 %)	0.013 (2.8 %)
95 % limits for *u*_cSH_	± 200 %	± 200 %	± 200 %

**Table 9:** Step height repeatability data grouped by test structure

	TS1	TS2	TS3	TS4
*n*	12	12	12	12
|*stepN*ABave| (in μm)	0.486	0.469	0.474	0.478
95 % limits for |*stepN*_AB_|	± 6.4 %	± 8.7 %	± 7.0 %	± 8.5 %
*u*_cave_ (in μm)	0.014 (2.8 %)	0.015 (3.1 %)	0.011 (2.4 %)	0.018 (3.7 %)
95 % limits for *u*_cSH_	± 28.3 %	± 71.6 %	± 27.7 %	± 81.2 %

**Table 10:** Interferometer pixel-to-pixel spacing requirements^a^

Magnification, ×	Pixel-to-pixel spacing, μm
5	< 1.57
10	< 0.83
20	< 0.39
40	< 0.21
80	< 0.11

a This table does not include magnifications at or less than 2.5 × for optical interferometry because the pixel-to-pixel spacings will be too large for this work and the possible introduction of a second set of interferometric fringes in the data set at these magnifications can adversely affect the data. Therefore, magnifications at or less than 2.5 × shall not be used with optical interferometry.

## Footnotes

3 In this paper, commercial equipment, instruments, or processes may be identified. This does not imply recommendation or endorsement by NIST, nor does it imply that the equipment, instruments, or processes are the best available for the purpose.

4 A 0° orientation implies that the length of the beam is parallel to the *x*-axis of the test chip, the axes of which are shown in Fig. 1 and again in Fig. 2, with the connection point of the cantilever having a smaller *x*-value than the *x*-values associated with the suspended portion of the cantilever.

5 By inserting the inputs into the correct locations on the appropriate NIST Web page (http://www.eeel.nist.gov/812/test-structures/MEMSCalculator.htm), the calculations can be performed on-line in a matter of seconds.

6 Squeeze film and other damping phenomena routinely lead to amplitude dependent resonance frequencies and shifts in the natural frequency of the system, which may limit the accuracy of the technique.

7 The damping may not be present in bulk-micromachining processes because it is dependent upon the depth of the cavity and the vicinity of the sides of the cavity to the beam. (The damping phenomena are expected to be present in surface micromachining processes without the use of a backside etch, unless the measurement is performed in a vacuum.)

8 Consult the List of Symbols for the nomenclature used for *platNXt* and *σ_platNXt_*.

9 Consult the List of Symbols.

10 Consult *platNrDt* in the List of Symbols for the nomenclature used in the subscript of *σ_platNrDt_*.

11 The term participant refers to a single data set from a unique combination of measurement setup and researcher. That is, a single researcher equipped with multiple, unique instruments (e.g., a dual beam vibrometer and a single beam vibrometer) or different forms of excitation (e.g., PZT and thermal excitation) could serve as multiple “participants” in this round robin.

12 Table 3 specifies the value of each of the uncertainty components comprising the 3*u*_c_*_E_* uncertainty bars for the repeatability data point corresponding to the first cantilever in Fig. 6 with a length of 300 μm.

13 Participant #2 provided stylus profilometer results, and that instrument did not provide standard deviation values; however it did provide average roughness values. Although this test method uses standard deviation values, in this analysis for that laboratory average roughness values were inserted into the data sheet for analysis as opposed to standard deviation values.

14 Table 4 specifies the value of each of the uncertainty components comprising the 3*u*_cSH_ uncertainty bars for the first repeatability data point plotted in Fig. 7 (for TS1 in quad 1).

15 The upper left hand step height test structure in a quad, such as shown in Fig. 4, is called test structure “1” and it has a 0° orientation. Test structure “2” (the upper right test structure) has a 270° orientation. Test structure “3” (the bottom right test structure) has a 180° orientation. And test structure “4” (the bottom left test structure) has a 90° orientation.

## 7. Appendix A. Instrument Specifications

An optical vibrometer, stroboscopic interferometer, or comparable instrument is required for Young’s modulus measurements, as specified in Sec. 7.1. For step height measurements, an optical interferometer or comparable instrument is used as specified in Sec. 7.2.

### 7.1 Instrument Specifications for Young’s Modulus Measurements

For Young’s modulus measurements, an optical vibrometer, stroboscopic interferometer, or an instrument comparable to one of these is required that is capable of non-contact measurements of surface motion. This section briefly describes the operation and specifications for a typical single beam laser vibrometer, a dual beam laser vibrometer, and a stroboscopic interferometer. The specifications can be applied to comparable instruments, if appropriate.

For a single beam laser vibrometer, a typical schematic is given in Fig. 9. A signal generator provides an excitation signal, which excites the sample via a piezoelectric transducer (PZT). The measurement beam is positioned to a scan point on the sample (by means of mirrors) and is reflected back. The reflected laser light interferes with the reference beam at the beam splitter (BS). A photodetector (PD) records the interference signal, converting it into an electrical signal. The frequency difference between the beams is proportional to the instantaneous velocity of the vibration parallel to the measurement beam. (The Bragg cell is instrumental in determining the sign of the velocity.) The velocity decoder provides a voltage proportional to the instantaneous velocity.

A dual beam laser vibrometer incorporates two beams. The measurement beam is positioned to a scan point on the sample (for example, positioned near the tip of a cantilever). The reference beam emanates from the beam splitter (BS) shown in Fig. 9 and is positioned to a second point on the sample (for example, positioned on the support region at the base of the cantilever). The two scattered beams optically combine at the beam splitter where the reference beam is used to directly eliminate any movement of the sample also experienced by the measurement beam.

For a stroboscopioc interferometer, a simplified schematic of a typical setup is shown in Fig. 10(a). When operated in the static mode, the interferometer is used to determine surface profiles. The incident light travels through the microscope objective to the beam splitter. Half of the light travels to the sample surface and then back to the beam splitter. The other half is reflected to a reference surface and then back to the beam splitter. These two paths of light recombine at the beam splitter to form interference light fringes. As the interferometer scans downward, an intensity envelope incorporating these fringes is determined by the software [see Fig. 10(b)]. The peak contrast of the fringes, phase, or both are used in determining the sample height at that pixel location. The surface profile is found by collecting sample height data for each pixel within the field of view (FOV). When operated in the dynamic mode, the incident light is strobed at the same frequency as that used to actuate the device. The sample is actuated, for example, after securing it to the top of a PZT. The phase, frequency, and drive signal to the strobe and PZT are varied, performing a downward scan as is done for static devices at each combination to obtain successive 3D images as the sample cycles through its range of motion.

Specifications for the (previous) above instruments or a comparable instrument are as follows:

1. The microscope objective or objectives should be chosen to allow for sufficient resolution of the cantilever or fixed-fixed beam and a portion of the surrounding sample. The objective(s) should have a FOV that can encompass at least half of the length of the cantilever or fixed-fixed beam being measured. Typically, a 4× and a 20× objective will suffice.
2. The signal generator should be able to produce a waveform function (such as a periodic chirp function^16^ or a sine wave function^17^) if applicable, such that from its use, a reproducible resonance frequency can be obtained and good 3-D oscillating images can be obtained such that it is obvious by inspection that the beam is in resonance.
3. The instrument shall be capable of producing a magnitude versus frequency plot from which the resonance frequency can be obtained.
4. The instrument should be capable of obtaining 3-D images of beam oscillations in order to ascertain if the correct frequency peak has been chosen as the beam’s resonance frequency.
5. An estimate for the maximum frequency of the instrument needed for a resonating cantilever, *f_caninit_*, is at least the value calculated using the following equation:^18^
  (51) $$f_{caninit}=\sqrt{\frac{E_{init}t^{2}}{38.330\rho L_{can}^{4}}},$$
  as derived in Sec. 8.1. An estimate for the maximum frequency of the instrument needed for a resonating fixed-fixed beam, *f_ffbinithi_*, is at least the value calculated using the following equation:
  (52) $$f_{\mathrm{ff}binithi}=\sqrt{\frac{E_{init}t^{2}}{0.946\rho L_{\mathrm{ff}b}^{4}}},$$
  as derived in Sec. 8.5.
6. An instrument that can make differential measurements (e.g., with the use of two laser beams) is recommended for use with fixed-fixed beams. It is also recommended for use with cantilevers, especially for estimated resonance frequencies less than 10 kHz and also if the value for *p_diff_* as calculated in the following equation is greater than or equal to 2 %:
  (53) $$p_{di\mathrm{ff}}=\left(1-\sqrt{1-\frac{1}{4Q^{2}}}\right)100\%.$$
  For a cantilever, the *Q*-factor, *Q*, in the above equation can be estimated using the following equation:
  (54) $$Q=\left[\frac{W_{can}\sqrt{E_{init}\rho }}{24\mu }\right]{\left(\frac{t}{L_{can}}\right)}^{2}.$$

### 7.2 Instrument Specifications for Step Height Measurements

For step height measurements, an optical interferometric microscope or comparable instrument is used that is capable of obtaining topographical 2-D data traces. (The stroboscopic interferometer operated in the static mode, as described in Sec. 7.1, can be used for these measurements.) Figure 11 is a schematic of a typical optical interferometric microscope that uses the method of coherence scanning interferometry [26], also called vertical scanning interferometry or scanning white light interferometry, for these measurements. However, any calibrated topography measuring instrument that has pixel-to-pixel spacings or sampling intervals as specified in Table 10 and that is capable of performing the test procedure with a vertical resolution less than 1 nm is permitted. The optical interferometric microscope or comparable instrument must be capable of measuring step heights to at least 5 μm higher than the step heights to be measured and must be capable of extracting standard deviations.

## 8. Appendix B. Supplemental Material for Young’s Modulus Measurements

This appendix contains supplemental material for Young’s modulus measurements for reference only. In Sec. 8.1, the derivation of the Young’s modulus equation from a resonating cantilever is presented. In Sec. 8.2, the residual stress and stress gradient equations are given. The equations for Young’s modulus measurements as obtained from a fixed-fixed beam are given in Sec. 8.3 followed by two derivations. The deriviation of the Young’s modulus equation from a resonating fixed-fixed beam assuming simply supported boundary conditions is given in Sec. 8.4, and the derivation of the Young’s modulus equation from a resonating fixed-fixed beam assuming clamped-clamped boundary conditions is given in Sec. 8.5. Lastly, Sec. 8.6 presents the calculation of *σ_fQ_* as discussed in Sec. 4.2.

### 8.1 Derivation of Young’s Modulus Equation From a Resonating Cantilever

In this section, the Young’s modulus equation is presented as derived, in a manner similar to that presented in [12], from measurements taken on a resonating cantilever. Clamped-free boundary conditions are assumed, as given by the following equations:

(55) $${\left(X\right)}_{x=0}=0,$$

(56) $${\left(\frac{dX}{dx}\right)}_{x=0}=0,$$

(57) $${\left(\frac{d^{2}X}{dx^{2}}\right)}_{x=L}=0,$$

and

(58) $${\left(\frac{d^{3}X}{dx^{3}}\right)}_{x=L}=0,$$

where the cantilever attachment point is at *x* = 0 μm, where the tip of the cantilever is at *x* = *L*, where *L* is the length of the cantilever, and where *X* is the normal function [12].

The equation of motion for all free transverse vibrations is given by the following equation [12]:

(59) $$\left(\frac{d^{4}X}{dx^{4}}\right)-\left(\frac{\rho A}{EI}\right)\omega^{2}X=0,$$

where *E*, *I*, *ρ*, *A*, and *ω* are the Young’s modulus, the area moment of inertia about the neutral axis, the density, the area, and the angular frequency, respectively. To help solve this fourth-order differential equation, the following notation is introduced:

(60) $$\omega =ak^{2}=k^{2}\sqrt{\frac{EI}{\rho A}},$$

such that the equation of motion can be given by the following equation:

(61) $$\left(\frac{d^{4}X}{dx^{4}}\right)-k^{4}X=0.$$

A solution to the above equation yields:

(62) $$\begin{array}{l} X=C_{1}(\cos\;kx+\cosh\;kx)+C_{2}(\cos\;kx-\cosh\;kx)\\ \;+C_{3}(\sin\;kx+\sinh\;kx)+C_{4}(\sin\;kx-\sinh\;kx). \end{array}$$

[This can be demonstrated by differentiating Eq. (62) four times and plugging the result into Eq. (61) along with Eq. (62) to achieve an identity.] Applying the four boundary conditions yields the following equation:

(63) $$\begin{array}{l} \left(\cos^{2}kL+\sin^{2}kL)+(\cosh^{2}kL-\sinh^{2}kL\right)\\ \;=-2\cos\;kL\cosh\;kL. \end{array}$$

However, since

(64) $$\cos^{2}x+\sin^{2}x=1$$

and

(65) $$\cosh^{2}x-\sinh^{2}x=1,$$

Equation (63) can be rewritten as

(66) $$\cos k_{i}L\cosh k_{i}L=-1,$$

where $k_{i}L\approx \left(i-\frac{1}{2}\right)\pi$with *i* = 1,2,3…∞ or *k_i_L* = 1.875, 4.694, 7.855,….

The frequency equation is given by:

(67) $$f_{i}=\frac{\omega_{i}}{2\pi }=\frac{ak_{i}^{2}}{2\pi },$$

where

(68) $$a=\sqrt{\frac{EI}{\rho A}}.$$

Therefore, for *k*_1_*L* = 1.875, we have

(69) $$f_{1}=\frac{a}{2\pi }\frac{k_{1}^{2}L^{2}}{L^{2}}=\frac{a}{2\pi }{\left(\frac{1.875}{L}\right)}^{2}$$

or

(70) $$f_{1}=\frac{{\left(1.875\right)}^{2}}{2\pi L^{2}}\sqrt{\frac{EI}{\rho A}}.$$

But

(71) $$I=\frac{1}{12}At^{2},$$

or rewritten

(72) $$\sqrt{\frac{I}{A}}=\frac{t}{\sqrt{12}},$$

where *t* is the thickness so

(73) $$f_{1}=\frac{{\left(1.875\right)}^{2}}{2\pi L^{2}}\frac{t}{\sqrt{12}}\sqrt{\frac{E}{\rho }}=0.16152\frac{t}{L^{2}}\sqrt{\frac{E}{\rho }}$$

and

(74) $$f_{1}=\sqrt{\frac{Et^{2}}{38.330\rho L^{4}}}$$

stated already as Eq. (1) in Sec. 3.1 and Eq. (51) in Sec. 7.1. Soving for *E* produces the following equation:

(75) $$E=\frac{38.330\rho f_{1}^{2}L^{4}}{t^{2}}$$

or Eq. (7) in Sec. 3.1.

### 8.2 Residual Stress and Stress Gradient Equations

Given the Young’s modulus value, *E*, obtained from Eq. (7) in Sec. 3.1, the residual stress, *σ*_r_, and the stress gradient, *σ*_g_, can be found from Eqs. (76) and (80) presented in this section.

To calculate residual stress, the residual strain of the thin film layer, *ε*_r_, must also be known. This value and its combined standard uncertainty value, *u_cεr_*, are found from measurements of a fixed-fixed beam test structure comprised of that layer using ASTM E2245. (ASTM E2245 can be consulted to determine if the fixed-fixed beam used for this residual strain measurement can also be used to find *f*_ffb_ and *E* in Sec. 8.3. However, it is recommended that the Young’s modulus value be obtained from the average resonance frequency of a cantilever as specified in Sec. 3.1 due to a lower resulting value for *u*_c_*_E_*.) Then, the residual stress is calculated using the following equation:

(76) $$\sigma_{r}=E\varepsilon_{r}.$$

The combined standard uncertainty for residual stress, *u_cσr_*, is then calculated using the folowing equation:

(77) $$u_{c\sigma r}=\sqrt{u_{E(\sigma r)}^{2}+u_{\varepsilon r(\sigma r)}^{2}},$$

where *u_E_*_(_*_σr_*_)_ and *u_ε_*_r(_*_σr_*_)_ are defined in the List of Symbols. In determining the combined standard uncertainty, a Type B evaluation [17] (i.e., one that uses means other than the statistical Type A analysis) is used for each source of error.

The uncertainty equation for *u_E_*_(_*_σr_*_)_ in the above equation is determined from the minimum and maximum residual stress values (namely, *σ*_rmin_ and *σ*_rmax_, respectively). Assuming a Gaussian distribution, *u_E_*_(_*_σr_*_)_ is calculated using the following equation:

(78) $$u_{E(\sigma r)}=u_{cE}\left|\varepsilon_{r}\right|,$$

where *u*_c_*_E_* is the combined standard uncertainty of the Young’s modulus measurement as given in Sec. 4.2 (or *u_E_* from Sec. 8.3 can be used although this is not recommended).

The uncertainty equation for *u_ε_*_r(σ_*_r_*_)_ in Eq. (77) is determined from the minimum and maximum residual stress values (namely, *σ*_rmin_ and *σ*_rmax_, respectively). Assuming a Gaussian distribution, *u_ε_*_r(_*_σr_*_)_ is calculated using the following equation:

(79) $$u_{\varepsilon r(\sigma r)}=u_{c\varepsilon r}E,$$

where *u*_c_*_εr_* was found in ASTM E2245 for residual strain.

To calculate stress gradient, the strain gradient of the thin film layer, *s*_g_, must also be known. This value and its combined standard uncertainty value, *u*_c_*_s_*_g_, are found from measurements of a cantilever test structure comprised of that layer using ASTM E2246. (ASTM E2246 can be consulted to determine if the cantilever used for this strain gradient measurement can also be used to find *f_can_* and *E* in Sec. 3.1.) Then, the stress gradient, *σ*_g_, is calculated using the following equation:

(80) $$\sigma_{g}=Es_{g}.$$

The combined standard uncertainty for stress gradient, *u*_c_*_σ_*_g_, is then calculated using the following equation:

(81) $$u_{c\sigma g}=\sqrt{u_{E(\sigma g)}^{2}+u_{sg(\sigma g)}^{2}},$$

where *u_E_*_(_*_σ_*_g)_ and *u*_sg(_*_σ_*_g)_ are defined in the List of Symbols. In determining the combined standard uncertainty, a Type B evaluation [17] (i.e., one that uses means other than the statistical Type A analysis) is used for each source of error.

The uncertainty equation for *u_E_*_(_*_σ_*_g)_ in the above equation is determined from the minimum and maximum stress gradient values (namely, *σ*_gmin_ and *σ*_gmax_, respectively). Assuming a Gaussian distribution, *u_E_*_(_*_σ_*_g)_ is calculated using the following equation:

(82) $$u_{E(\sigma g)}=u_{cE}s_{g},$$

where *u*_c_*_E_* is the combined standard uncertainty of the Young’s modulus measurement as given in Sec. 4.2 (or *u_E_* from Sec. 8.3 can be used although this is not recommended).

The uncertainty equation for *u*_sg(_*_σ_*_g)_ in Eq. (81) is determined from the minimum and maximum stress gradient values (namely, *σ*_gmin_ and *σ*_gmax_, respectively). Assuming a Gaussian distribution, *u*_sg(_*_σ_*_g)_ is calculated using the following equation:

(83) $$u_{sg(\sigma g)}=u_{csg}E,$$

where *u*_csg_ was found in ASTM E2246 for strain gradient.

### 8.3 Young’s Modulus Measurements From Fixed-Fixed Beams

The Young’s modulus of a single layer can be obtained from resonance frequency measurements from a fixed-fixed beam comprised of the same material (although the use of a cantilever is recommended as described in Sec. 3.1 due to a lower resulting combined standard uncertainty value).

To determine an estimate for the resonance frequency of a fixed-fixed beam, the lower and upper bounds for the resonance frequency (namely, *f_ffbinitlo_* and *f_ffbinithi_*, respectively) are calculated using the following equations (as derived in Sec. 8.4 and Sec. 8.5, respectively):^19^

(84) $$f_{\mathrm{ff}binitlo}=\sqrt{\frac{E_{init}t^{2}}{4.864\rho L_{\mathrm{ff}b}^{4}}}$$

and

(85) $$f_{\mathrm{ff}binithi}=\sqrt{\frac{E_{init}t^{2}}{0.946\rho L_{\mathrm{ff}b}^{4}}},$$

where *E_init_* is the initial estimate for the Young’s modulus value of the thin film layer, *t* is the thickness of the suspended layer, *ρ* is the density, and *L_ffb_* is the suspsended length of the fixed-fixed beam.

Measurements are taken which encompass the average of *f_ffbinitlo_* and *f_ffbinithi_* and an excitation-magnitude versus frequency plot is obtained from which the resonance frequency is found. For a given fixed-fixed beam, given the average of at least three resonance frequency measurements, *f_ffb_*, two values of Young’s modulus are calculated; one assuming simply supported boundary conditions at both supports, *E_simple_*, (as derived in Sec. 8.4) and given below:

(86) $$E_{simple}=\frac{48\pi^{2}}{\pi^{4}}\frac{\rho f_{\mathrm{ff}b}^{2}L_{\mathrm{ff}b}^{4}}{t^{2}}=\frac{4.864\rho f_{\mathrm{ff}b}^{2}L_{\mathrm{ff}b}^{4}}{t^{2}}$$

and one assuming clamped-clamped boundary conditions, *E_clamped_*, (as derived in Sec. 8.5) and given below:

(87) $$E_{clamped}=\frac{48\pi^{2}}{4.730^{4}}\frac{\rho f_{\mathrm{ff}b}^{2}L_{\mathrm{ff}b}^{4}}{t^{2}}=\frac{0.946\rho f_{\mathrm{ff}b}^{2}L_{\mathrm{ff}b}^{4}}{t^{2}}.$$

Given these two values, *E_simple_* and *E_clamped_*, the average Young’s modulus is calculated using the following equation:

(88) $$E=\frac{E_{simple}+E_{clamped}}{2}$$

and recorded as the Young’s modulus value, *E*, for the thin film layer.

The standard uncertainty, *u_E_*, for the Young’s modulus of the film is determined from a Type B evaluation [17] using the calculated minimum and maximum Young’s modulus values (namely, *E_clamped_* and *E_simple_*, respectively). With 99.7 % confidence, assuming a Gaussian distribution, the value of *E* lies between *E_clamped_* and *E_simple_*. Therefore, *u_E_* is calculated as follows:

(89) $$u_{E}=\frac{E_{simple}-E_{clamped}}{6}=\frac{3.918\rho f_{\mathrm{ff}b}^{2}L_{\mathrm{ff}b}^{4}}{t^{2}}.$$

### 8.4 Derivation of Young’s Modulus Equation From a Resonating Fixed-Fixed Beam Assuming Simply Supported Boundary Conditions

In this section, the Young’s modulus equation is derived, in a manner similar to that presented in [12], from resonance frequency measurements taken on a fixed-fixed beam. Simply supported boundary conditions are assumed for both supports, as given by the following equations:

(90) $${\left(X\right)}_{x=0}=0,$$

(91) $${\left(\frac{d^{2}X}{dx^{2}}\right)}_{x=0}=0,$$

(92) $${\left(X\right)}_{x=L}=0,$$

and

(93) $${\left(\frac{d^{2}X}{dx^{2}}\right)}_{x=L}=0,$$

where the fixed-fixed beam attachment points are at *x* = 0 μm and at *x* = *L*, where *L* is the length of the fixed-fixed beam, and where *X* is the normal function [12].

The equation of motion for all free transverse vibrations is given by the following equation [12]:

(94) $$\left(\frac{d^{4}X}{dx^{4}}\right)-\left(\frac{\rho A}{EI}\right)\omega^{2}X=0,$$

where *E*, *I*, *ρ*, *A*, and *ω* are the Young’s modulus, the area moment of inertia about the neutral axis, the density, the area, and the angular frequency, respectively. To help solve this fourth-order differential equation, the following notation is introduced:

(95) $$\omega =ak^{2}=k^{2}\sqrt{\frac{EI}{\rho A}},$$

such that the equation of motion can be given by the following equation:

(96) $$\left(\frac{d^{4}X}{dx^{4}}\right)-k^{4}X=0.$$

A solution to the above equation yields:

(97) $$\begin{array}{l} X=C_{1}(\cos\;kx+\cosh\;kx)+C_{2}(\cos\;kx-\cosh\;kx)\\ \;+C_{3}(\sin\;kx+\sinh\;kx)+C_{4}(\sin\;kx-\sinh\;kx). \end{array}$$

[This can be demonstrated by differentiating Eq. (97) four times and plugging the result into Eq. (96) along with Eq. (97) to achieve an identity.] Applying the four boundary conditions yields the following equation:

(98) $$\begin{array}{c} \sin\;k_{i}L=0,\\ where\;k_{i}=\frac{i\pi }{L}\;with\;i=1,2,3,\ldots ,\infty . \end{array}$$

The frequency equation is given by:

(99) $$f_{i}=\frac{\omega_{i}}{2\pi }=\frac{ak_{i}^{2}}{2\pi },$$

where

(100) $$a=\sqrt{\frac{EI}{\rho A}}.$$

Therefore, with *k*_1_*L = π* we have

(101) $$f_{1}=\frac{a}{2\pi }\frac{k_{1}^{2}L^{2}}{L^{2}}=\frac{a}{2\pi }{\left(\frac{\pi }{L}\right)}^{2}=\frac{a\pi }{2L^{2}}$$

or

(102) $$f_{1}=\frac{\pi }{2L^{2}}\sqrt{\frac{EI}{\rho A}}.$$

But

(103) $$I=\frac{1}{12}At^{2},$$

or rewritten

(104) $$\sqrt{\frac{I}{A}}=\frac{t}{\sqrt{12}},$$

where *t* is the thickness so

(105) $$f_{1}=\frac{\pi }{2L^{2}}\frac{t}{\sqrt{12}}\sqrt{\frac{E}{\rho }}=\frac{\pi }{2\sqrt{12}}\sqrt{\frac{Et^{2}}{\rho L^{4}}}$$

and

(106) $$f_{1}=\sqrt{\frac{Et^{2}}{4.864\rho L^{4}}}$$

[see Eq. (84) in Sec. 8.3]. Solving for *E* produces the following:

(107) $$E=\frac{4.864\rho f_{1}^{2}L^{4}}{t^{2}}$$

[see Eq. (86) in Sec. 8.3].

### 8.5. Derivation of Young’s Modulus Equation From a Resonating Fixed-Fixed Beam Assuming Clamped-Clamped Boundary Conditions

In this section, the Young’s modulus equation is derived, in a manner similar to that presented in [12], from resonance frequency measurements taken on a fixed-fixed beam. Clamped-clamped boundary conditions are assumed, as given by the following equations:

(108) $${\left(X\right)}_{x=0}=0,$$

(109) $${\left(\frac{dX}{dx}\right)}_{x=0}=0,$$

(110) $${\left(X\right)}_{x=L}=0,$$

and

(111) $${\left(\frac{dX}{dx}\right)}_{x=L}=0,$$

where the fixed-fixed beam attachment points are at *x* = 0 μm and at *x* = *L*, where *L* is the length of the fixed-fixed beam, and where *X* is the normal function [12].

The equation of motion for all free transverse vibrations is given by the following equation [12]:

(112) $$\left(\frac{d^{4}X}{dx^{4}}\right)-\left(\frac{\rho A}{EI}\right)\omega^{2}X=0,$$

where *E*, *I*, *ρ*, *A*, and *ω* are the Young’s modulus, the area moment of inertia about the neutral axis, the density, the area, and the angular frequency, respectively. To help solve this fourth-order differential equation, the following notation is introduced:

(113) $$\omega =ak^{2}=k^{2}\sqrt{\frac{EI}{\rho A}},$$

such that the equation of motion can be given by the following equation:

(114) $$\left(\frac{d^{4}X}{dx^{4}}\right)-k^{4}X=0.$$

A solution to the above equation yields:

(115) $$\begin{array}{l} X=C_{1}(\cos\;kx+\cosh\;kx)+C_{2}(\cos\;kx-\cosh\;kx)\\ \;+C_{3}(\sin\;kx+\sinh\;kx)+C_{4}(\sin\;kx-\sinh\;kx). \end{array}$$

[This can be demonstrated by differentiating Eq. (115) four times and plugging the result into Eq. (114) along with Eq. (115) to achieve an identity.] Applying the four boundary conditions yields the following equation:

(116) $$\begin{array}{l} \left(\cos^{2}kL+\sin^{2}kL)+(\cosh^{2}kL-\sinh^{2}kL\right)\\ \;=2\cos\;kL\;\cosh\;kL. \end{array}$$

However, since

(117) $$\cos^{2}x+\sin^{2}x=1$$

and

(118) $$\cosh^{2}x-\sinh^{2}x=1,$$

Eq. (116) can be rewritten as

(119) $$\begin{array}{c} \cos\;k_{i}L\cosh\;k_{i}L=1,\\ \text{where}\;k_{i}L\approx \left(i+\frac{1}{2}\right)\pi \;\text{with}\;i=1,2,3,\ldots ,\infty \\ \text{or}\;k_{i}L=4.730,\;7.853,\;10.996\ldots . \end{array}$$

The frequency equation is given by:

(120) $$f_{i}=\frac{\omega_{i}}{2\pi }=\frac{ak_{i}^{2}}{2\pi },$$

where

(121) $$a=\sqrt{\frac{EI}{\rho A}}.$$

Therefore, for *k_1_L* = 4.730, we have

(122) $$f_{1}=\frac{a}{2\pi }\frac{k_{1}^{2}L^{2}}{L^{2}}=\frac{a}{2\pi }{\left(\frac{4.730}{L}\right)}^{2}$$

or

(123) $$f_{1}=\frac{{\left(4.730\right)}^{2}}{2\pi L^{2}}\sqrt{\frac{EI}{\rho A}}.$$

But

(124) $$I=\frac{1}{12}At^{2},$$

or rewritten

(125) $$\sqrt{\frac{I}{A}}=\frac{t}{\sqrt{12}},$$

where *t* is the thickness so

(126) $$f_{1}=\frac{{\left(4.730\right)}^{2}}{2\pi L^{2}}\frac{t}{\sqrt{12}}\sqrt{\frac{E}{\rho }}=1.0279\frac{t}{L^{2}}\sqrt{\frac{E}{\rho }}$$

and

(127) $$f_{1}=\sqrt{\frac{Et^{2}}{0.946\rho L^{4}}},$$

which agrees with Eq. (52) in Sec. 7.1 and Eq. (85) in Sec. 8.3. Solving for *E* produces the following:

(128) $$E=\frac{0.946\rho f_{1}^{2}L^{4}}{t^{2}},$$

which agrees with Eq. (87) in Sec. 8.3.

### 8.6 Calculation of *σ_fQ_*

To obtain *σ_fQ_* (as given in Sec. 4.2), the following equation is used:

(129) $$\sigma_{fQ}=\sqrt{u_{W(f)}^{2}+u_{L(f)}^{2}+u_{\rho (f)}^{2}+u_{t(f)}^{2}+u_{E(f)}^{2}+u_{\mu (f)}^{2}},$$

where *u_W(f)_*, *u_L(f)_*, *u_ρ(f)_*, *u_t(f)_*, *u_E(f)_*, and *u_μ(f)_* are found below.

The uncertainty equation for *u_W(f)_* is determined from a calculation of the potential variation in the undamped resonance frequencies due to the uncertainty in the cantilever width, *W_can_*, as given below:

(130) $$f_{undamped\;\min}=\frac{f_{dampedave}}{\sqrt{1-\frac{1}{4{\left[\frac{(W_{can}+3\sigma_{W})\sqrt{E\rho }}{24\mu }{\left(\frac{t}{L_{can}}\right)}^{2}\right]}^{2}}}}$$

(131) $$f_{undamped\;\max}=\frac{f_{dampedave}}{\sqrt{1-\frac{1}{4{\left[\frac{(W_{can}-3\sigma_{W})\sqrt{E\rho }}{24\mu }{\left(\frac{t}{L_{can}}\right)}^{2}\right]}^{2}}}},$$

where *σ_W_* is the estimated one sigma uncertainty of the value of *W_can_* and *f_dampedave_* is the average of the three damped resonance frequency values (*f_damped_*_1_, *f_damped_*_2_, and *f_damped_*_3_). With 99.7 % confidence, assuming a Gaussian distribution (and assuming *u_L(f)_*, *u_ρ(f)_*, *u_t(f)_*, *u_E(f)_*, and *u_μ(f)_* equal zero), the value for *f_undamped_* lies between *f_undamped_*_min_ and *f_undamped_*_max_. Therefore, *u_W(f)_* is calculated as follows:

(132) $$u_{W(f)}=\frac{f_{undamped\;\max}-f_{undamped\;\min}}{6}.$$

The uncertainty equation for *u_L(f)_* is determined from the minimum and maximum undamped resonance frequencies (namely, *f_undamped_*_min_ and *f_undamped_*_max_, respectively) as given below:

(133) $$f_{undamped\;\min}=\frac{f_{dampedave}}{\sqrt{1-\frac{1}{4{\left[\frac{W_{can}\sqrt{E\rho }}{24\mu }{\left(\frac{t}{\left(L_{can}-3\sigma_{L}\right)}\right)}^{2}\right]}^{2}}}}$$

(134) $$f_{undamped\;\max}=\frac{f_{dampedave}}{\sqrt{1-\frac{1}{4{\left[\frac{W_{can}\sqrt{E\rho }}{24\mu }{\left(\frac{t}{\left(L_{can}+3\sigma_{L}\right)}\right)}^{2}\right]}^{2}}}},$$

where *σ_L_* is the estimated one sigma uncertainty of the value of *L_can_*. With 99.7 % confidence, assuming a Gaussian distribution (and assuming *u_W(f)_*, *u_ρ(f)_*, *u_t(f)_*, *u_E(f)_*, and *u_μ(f)_* equal zero), the value for *f_undamped_* lies between *f_undamped_*_min_ and *f_undamped_*_max_. Therefore, *u_L(f)_* is calculated as follows:

(135) $$u_{L(f)}=\frac{f_{undamped\;\max}-f_{undamped\;\min}}{6}.$$

The uncertainty equation for *u_ρ(f)_* is determined from the minimum and maximum undamped resonance frequencies (namely, *f_undamped_*_min_ and *f_undamped_*_max_, respectively) as given below:

(136) $$f_{undamped\;\min}=\frac{f_{dampedave}}{\sqrt{1-\frac{1}{4{\left[\frac{W_{can}\sqrt{E(\rho +3\sigma_{\rho })}}{24\mu }{\left(\frac{t}{L_{can}}\right)}^{2}\right]}^{2}}}}$$

(137) $$f_{undamped\;\max}=\frac{f_{dampedave}}{\sqrt{1-\frac{1}{4{\left[\frac{W_{can}\sqrt{E(\rho -3\sigma_{\rho })}}{24\mu }{\left(\frac{t}{L_{can}}\right)}^{2}\right]}^{2}}}}.$$

where *σ_ρ_* is the estimated one sigma uncertainty of the value of *ρ*. With 99.7 % confidence, assuming a Gaussian distribution (and assuming *u_W(f)_*, *u_L(f)_*, *u_t(f)_*, *u_E(f)_*, and *u_μ(f)_* equal zero), the value for *f_undamped_* lies between *f_undamped_*_min_ and *f_undamped_*_max_. Therefore, *u_ρ(f)_* is calculated as follows:

(138) $$u_{\rho (f)}=\frac{f_{undamped\;\max}-f_{undamped\;\min}}{6}.$$

The uncertainty equation for *u_t(f)_* is determined from the minimum and maximum undamped resonance frequencies (namely, *f_undamped_*_min_ and *f_undamped_*_max_, respectively) as given below:

(139) $$f_{undamped\;\min}=\frac{f_{dampedave}}{\sqrt{1-\frac{1}{4{\left[\frac{W_{can}\sqrt{E\rho }}{24\mu }{\left(\frac{t+3\sigma_{thick}}{L_{can}}\right)}^{2}\right]}^{2}}}}$$

(140) $$f_{undamped\;\max}=\frac{f_{dampedave}}{\sqrt{1-\frac{1}{4{\left[\frac{W_{can}\sqrt{E\rho }}{24\mu }{\left(\frac{t-3\sigma_{thick}}{L_{can}}\right)}^{2}\right]}^{2}}}},$$

where *σ_thick_* is the one sigma uncertainty of the value of *t*, which is found using the electro-physical technique [18]. Data Sheet T.1 [10] can be used to calculate *σ_thick_*. With 99.7 % confidence, assuming a Gaussian distribution (and assuming *u_W(f)_*, *u_L(f)_*, *u_ρ(f)_*, *u_E(f)_*, and *u_μ(f)_* equal zero), the value for *f_undamped_* lies between *f_undamped_*_min_ and *f_undamped_*_max_. Therefore, *u_t(f)_* is calculated as follows:

(141) $$u_{t(f)}=\frac{f_{undamped\;\max}-f_{undamped\;\min}}{6}.$$

The uncertainty equation for *u_E(f)_* is determined from the minimum and maximum undamped resonance frequencies (namely, *f_undamped_*_min_ and *f_undamped_*_max_, respectively) as given below:

(142) $$f_{undamped\;\min}=\frac{f_{dampedave}}{\sqrt{1-\frac{1}{4{\left[\frac{W_{can}\sqrt{(E+3u_{cE0})\rho }}{24\mu }{\left(\frac{t}{L_{can}}\right)}^{2}\right]}^{2}}}}$$

(143) $$f_{undamped\;\max}=\frac{f_{dampedave}}{\sqrt{1-\frac{1}{4{\left[\frac{W_{can}\sqrt{(E-3u_{cE0})\rho }}{24\mu }{\left(\frac{t}{L_{can}}\right)}^{2}\right]}^{2}}}},$$

where *u_cE_*_0_ is given by:

(144) $$u_{cE0}=\sqrt{u_{thick}^{2}+u_{\rho }^{2}+u_{L}^{2}+u_{freq}^{2}+u_{fresol}^{2}}.$$

With 99.7 % confidence, assuming a Gaussian distribution (and assuming *u_W(f)_*, *u_L(f)_*, *u_ρ(f)_*, *u_t(f)_*, and *u_μ(f)_* equal zero), the value for *f_undamped_* lies between *f_undamped_*_min_ and *f_undamped_*_max_. Therefore, *u_E(f)_* is calculated as follows:

(145) $$u_{E(f)}=\frac{f_{undamped\;\max}-f_{undamped\;\min}}{6}.$$

The uncertainty equation for *u_μ(f)_* is determined from the minimum and maximum undamped resonance frequencies (namely, *f_undamped_*_min_ and *f_undamped_*_max_, respectively) as given below:

(146) $$f_{undamped\;\min}=\frac{f_{dampedave}}{\sqrt{1-\frac{1}{4{\left[\frac{W_{can}\sqrt{E\rho }}{24(\mu -3\sigma_{\mu })}{\left(\frac{t}{L_{can}}\right)}^{2}\right]}^{2}}}}$$

(147) $$f_{undamped\;\max}=\frac{f_{dampedave}}{\sqrt{1-\frac{1}{4{\left[\frac{W_{can}\sqrt{E\rho }}{24(\mu +3\sigma_{\mu })}{\left(\frac{t}{L_{can}}\right)}^{2}\right]}^{2}}}},$$

where *σ_μ_* is the estimated one sigma uncertainty of the value of *μ*. With 99.7 % confidence, assuming a Gaussian distribution (and assuming *u_W(f)_*, *u_L(f)_*, *u_ρ(f)_*, *u_t(f)_*, and *u_E(f)_* equal zero), the value for *f_undamped_* lies between *f_undamped_*_min_ and *f_undamped_*_max_. Therefore, *u_μ(f)_* is calculated as follows:

(148) $$u_{\mu (f)}=\frac{f_{undamped\;\max}-f_{undamped\;\min}}{6}.$$

## 9. Appendix C. Supplemental Material for Step Height Measurements

This appendix contains supplemental material for step height measurements. In Sec. 9.1, equations are presented for step height measurements taken from two step height test structures. In Sec. 9.2, the combined standard uncertainty equation is presented for this measurement. And, Sec. 9.3 presents the derivation of *u_Lstep_*, a component in the combined standard uncertainty calculation for a step height measurement taken from one step height test structure, as indicated in Sec. 4.3.

### 9.1 Step Height Measurements From Two Step Height Test Structures

Step height measurements can be taken from either one or two step height test structures. To obtain a step height measurement from one step height test structure (the recommended approach), consult Sec. 3.2. This current section presents the equations for a step height measurement from two step height test structures on the same chip.

To determine a step height measurement from two step height test structures on the same chip, the following three calculations are made: (1) a calculation of the reference platform height for both step height test structures, which assumes that the reference platforms are comprised of the same layers, (2) a calculation of the two platform heights involved in the step, and (3) a calculation of the step height.

The first calculation is of the reference platform height for both of the step height test structures. To calculate the reference platform height, such as *plat*1r from test structure “1,” the following equations are used:

(149) $$plat1\text{rW}=\frac{\left(plat1\text{rWa}+plat1\text{rWb}+plat1\text{rWc}\right)}{3},$$

(150) $$plat1\text{rE}=\frac{\left(plat1\text{rEa}+plat1\text{rEb}+plat1\text{rEc}\right)}{3},$$

and

(151) $$plat1r=\frac{\left(plat1\text{rW}+plat1\text{rE}\right)}{2},$$

where *platN*r*D*, *platN*r*Dt*, and *platNX* in the List of Symbols provide details associated with the nomenclature used. Similarly, to calculate the reference platform height, such as *plat*2r from test structure “2,” the following equations are used:

(152) $$plat2\text{rW}=\frac{\left(plat2\text{rWa}+plat2\text{rWb}+plat2\text{rWc}\right)}{3},$$

(153) $$plat2\text{rE}=\frac{\left(plat2\text{rEa}+plat2\text{rEb}+plat2\text{rEc}\right)}{3},$$

and

(154) $$plat2r=\frac{\left(plat2\text{rW}+plat2\text{rE}\right)}{2}.$$

The second calculation is of the two platform heights (namely, *platNX* and *platMY*) involved in the step. The following equations are used:

(155) $$platNX=\frac{\left(platNXa+platNXb+platNXc\right)}{3}-platNr$$

and

(156) $$platMY=\frac{\left(platMYa+platMYb+platMYc\right)}{3}-platMr,$$

where *platNXt* in the List of Symbols provides details associated with the nomenclature used. Therefore, for platform “A” in test structure “1” and platform “B” in test structure “2,” the corresponding platform heights (namely, *plat*1A and *plat*2B) are calculated using the following equations:

(157) $$plat1A=\frac{\left(plat1\text{Aa}+plat1\text{Ab}+plat1\text{Ac}\right)}{3}-plat1r$$

and

(158) $$plat2B=\frac{\left(plat2\text{Ba}+plat2\text{Bb}+plat2\text{Bc}\right)}{3}-plat2r.$$

The third calculation is of the step height, *stepN_X_M_Y_*, where the List of Symbols provides details associated with the nomenclature. The following equation is used:

(159) $$stepN_{X}M_{Y}=platMY-platNX,$$

which for a step height from platform “A” in test stucture “1” to platform “B” in test structure “2” (or *step*1_A_2_B_) becomes the following:

(160) $$step1_{A}2_{B}=plat2B-plat1A.$$

The combined standard uncertainty equation for the above calculation is given in Sec. 9.2.

### 9.2 Uncertainty Calculation When Two Step Height Test Structures Are Used in a Step Height Measurement

If two step height test structures on the same chip are used to obtain a step height measurement, such as *stepN_X_M_Y_* from *platNX* and *platMY* as given in Eq. (159) in Sec. 9.1, the following equation is used to calculate the combined standard uncertainty, *u*_cSH_ [17]:

(161) $$u_{c\text{SH}}=\sqrt{u_{platNX}^{2}+u_{platMY}^{2}},$$

where *u_platNX_* is given in the List of Symbols and is found using the following equation:

(162) $$\begin{array}{l} u_{platNX}=\\ \sqrt{u_{LplatNX}^{2}+u_{WplatNX}^{2}+u_{cert}^{2}+u_{repeat}^{2}+u_{drift}^{2}+u_{linear}^{2}}, \end{array}$$

where *u_LplatNX_*, *u_WplatNX_*, *u_cert_*, *u*_repeat_, *u*_drift_, and *u*_linear_ are given in the List of Symbols and discussed in the paragraphs that follow and where *u_platMY_* in Eq. (161) is found by replacing occurrences of *N* with *M* and occurrences of *X* with *Y*, as appropriate, in Eq. (162) and other applicable calculations. In determining the combined standard uncertainty, a Type B evaluation [17] (i.e., one that uses means other than the statistical Type A analysis) is used for each source of error, except where noted.

The uncertainty equation for *u_LplatNX_* is determined from the minimum and maximum platform height values (namely, *platNX*_min_ and *platNX*_max_, respectively) as given below:

(163) $$platNX_{\min}=\left|platNX\right|-3\sigma_{LplatNX}$$

and

(164) $$platNX_{\max}=\left|platNX\right|+3\sigma_{LplatNX},$$

where *σ_LplatNX_*, as derived within Sec. 9.3, is given by:

(165) $$\sigma_{LplatNX}=\sqrt{{\left(\sigma_{platNX\text{ave}}-\sigma_{\text{rough}NX}\right)}^{2}}=\sigma_{platNX\text{ave}}-\sigma_{\text{rough}NX},$$

where *σ_platNX_*_ave_ is calculated using the following equation:

(166) $$\sigma_{platNX\text{ave}}=\frac{\sigma_{platNXa}+\sigma_{platNXb}+\sigma_{platNXc}}{3},$$

and where *σ*_rough_*_NX_* is the smallest of all the calibrated values obtained for *σ_platNXt_*. However, if the surfaces of *platNX*, *platMY*, *platN*r, and *platM*r all have identical compositions, then *σ*_rough_*_NX_* equals *σ*_rough_*_MY_*, which equals the smallest of all the values obtained for *σ_platNXt_*, *σ_platMYt_*, *σ_platN_*_r_*_Dt_*, and *σ_platM_*_r_*_Dt_*. With 99.7 % confidence, assuming a Gaussian distribution (and assuming *u_WplatNX_*, *u_cert_*, *u*_repeat_, *u*_drift_, and *u*_linear_ equal zero), the value for |*platNX*| lies between *platNX*_min_ and *platNX*_max_. Therefore, *u_LplatNX_* is calculated as follows:

(167) $$u_{LplatNX}=\frac{platNX_{\max}-platNX_{\min}}{6},$$

which simplifies to the following equation:

(168) $$u_{LplatNX}=\sigma_{LplatNX}=\sigma_{platNXave}-\sigma_{roughNX}.$$

The uncertainty equation for *u_WplatNX_* is determined from the minimum and maximum platform height values (namely, *platNX*_min_ and *platNX*_max_, respectively) as given below:

(169) $$platNX_{\min}=\left|platNX\right|-3\sigma_{WplatNX}$$

and

(170) $$platNX_{\max}=\left|platNX\right|+3\sigma_{WplatNX},$$

where *σ_WplatNX_* is given by the following equation:

(171) $$\sigma_{WplatNX}=\sqrt{s_{platNX}^{2}+s_{platNr}^{2}},$$

where *s_platNX_* is the standard deviation of the platform measurements (namely, *platNX*a, *platNX*b, and *platNX*c) from the data traces and *s_platN_*_r_ is the standard deviation of the measurements used in the calculation of the reference platform, *platN*r. With 99.7 % confidence, assuming a Gaussian distribution (and assuming *u_LplatNX_*, *u_cert_*, *u*_repeat_, *u*_drift_, and *u*_linear_ equal zero), the value for |*platNX*| lies between *platNX*_min_ and *platNX*_max_. Therefore, *u_WplatNX_* is calculated as follows:

(172) $$u_{WplatNX}=\frac{platNX_{\max}-platNX_{\min}}{6},$$

which simplifies to the following equation:

(173) $$u_{WplatNX}=\sigma_{WplatNX}=\sqrt{s_{platNX}^{2}+s_{platN\;r}^{2}}.$$

Therefore, the following equation is used to find *u_Wplat_*_1A_ for *plat*1A:

(174) $$u_{Wplat1A}=\sigma_{Wplat1A}=\sqrt{s_{plat1A}^{2}+s_{plat1r}^{2}},$$

where *s_plat_*_1A_ is the standard deviation of the measurements *plat*1Aa, *plat*1Ab, and *plat*1Ac and where *s_plat_*_1r_ is the standard deviation of the measurements (namely, *plat*1rWa, *plat*1rWb, *plat*1rWc, *plat*1rEa, *plat*1rEb, and *plat*1rEc) used to find *plat*1r. This component, *uWplat*1A, can be considered a Type A component.

The uncertainty equation for *u_cert_*, is determined from the minimum and maximum platform height values (namely, *platNX*_min_ and *platNX*_max_, respectively) as given:

(175) $$platNX_{\min}=\left|platNX\right|-3\left|platNX\right|\frac{\sigma_{cert}}{cert}$$

and

(176) $$platNX_{\max}=\left|platNX\right|+3\left|platNX\right|\frac{\sigma_{cert}}{cert}.$$

The uncertainty of the platform height is assumed to scale linearly with height. With 99.7 % confidence, assuming a Gaussian distribution (and assuming *u_LplatNX_*, *u_WplatNX_*, *u*_repeat_, *u*_drift_, and *u*_linear_ equal zero), the value for | *platNX* | lies between *platNX*_min_ and *platNX*_max_. Therefore, *u_cert_* is calculated as follows:

(177) $$u_{cert}=\frac{platNX_{\max}-platNX_{\min}}{6},$$

which simplifies to the following equation:

(178) $$u_{cert}=\frac{\sigma_{cert}}{cert}\left|platNX\right|.$$

The above equation is comparable to Eq. (39) after replacing |*stepN_XY_*| with |*platNX*|.

The uncertainty equation for *u*_repeat_ is determined from the minimum and maximum platform height values (namely, *platNX*_min_ and *platNX*_max_, respectively) as given below:

(179) $$platNX_{\min}=\left|platNX\right|-\left|platNX\right|\frac{z_{\text{repeat}}}{2{\bar{z}}_{6}}$$

and

(180) $$platNX_{\max}=\left|platNX\right|+\left|platNX\right|\frac{z_{\text{repeat}}}{2{\bar{z}}_{6}}.$$

The uncertainty of the platform height is assumed to scale linearly with height. Assuming a uniform distribution (and assuming *u_LplatNX_*, *u_WplatNX_*, *u_cert_*, *u*_drift_, and *u*_linear_ equal zero), the value for |*platNX*| lies between *platNX*_min_ and *platNX*_max_. Therefore, *u*_repeat_ is calculated as follows:

(181) $$u_{\text{repeat}}=\frac{platNX_{\max}-platNX_{\min}}{2\sqrt{3}}$$

which simplifies to the following equation:

(182) $$u_{repeat}=\frac{z_{repeat}}{2\sqrt{3}{\bar{z}}_{6}}\left|platNX\right|.$$

The above equation is comparable to Eq. (43) after replacing | *stepN_XY_*| with | *platNX* |.

The uncertainty equation for *u*_drift_ is determined from the minimum and maximum platform height values (namely, *platNX*_min_ and *platNX*_max_, respectively) as given below:

(183) $$platNX_{\min}=\left|platNX\right|-\left|platNX\right|\frac{z_{\text{drift}}}{2\bar{z}}$$

and

(184) $$platNX_{\max}=\left|platNX\right|+\left|platNX\right|\frac{z_{\text{drift}}}{2\bar{z}}.$$

The uncertainty of the platform height is assumed to scale linearly with height. Assuming a uniform distribution (and assuming *u_LplatNX_*, *u_WplatNX_*, *u_cert_*, *u*_repeat_, and *u*_linear_ equal zero), the value for | *platNX*| lies between *platNX*_min_ and *platNX*_max_. Therefore, *u*_drift_ is calculated as follows:

(185) $$u_{\text{drift}}=\frac{platNX_{\max}-platNX_{\min}}{2\sqrt{3}},$$

which simplifies to the following equation:

(186) $$u_{\text{drift}}=\frac{z_{\text{drift}}}{2\sqrt{3}\bar{z}}\left|platNX\right|.$$

The above equation is comparable to Eq. (47) after replacing |*stepN_XY_*| with |*platNX*|.

The uncertainty equation for *u*_linear_ is determined from the minimum and maximum platform height values (namely, *platNX*_min_ and *platNX*_max_, respectively) as given below:

(187) $$platNX_{\min}=\left|platNX\right|-\left|platNX\right|z_{\text{perc}}$$

and

(188) $$platNX_{\max}=\left|platNX\right|+\left|platNX\right|z_{\text{perc}}.$$

The uncertainty of the platform height is assumed to scale linearly with height. Assuming a uniform distribution (and assuming *u_LplatNX_*, *u_WplatNX_*, *u_cert_*, *u*_repeat_, and *u*_drift_ equal zero), the value for |*platNX*| lies between *platNX*_min_ and *platNX*_max_. Therefore, *u*_linear_ is calculated as follows:

(189) $$u_{\text{linear}}=\frac{platNX_{\max}-platNX_{\min}}{2\sqrt{3}},$$

which simplifies to the following equation:

(190) $$u_{\text{linear}}=\frac{z_{\text{perc}}}{\sqrt{3}}\left|platNX\right|.$$

The above equation is comparable to Eq. (50) after replacing |*stepN_XY_*| with |*platNX*|.

### 9.3 Derivation of *u_Lstep_*

As mentioned in Sec. 4.3, one component of the combined standard uncertainty equation for a step height measurement taken from one step height test structure is *u_Lstep_*, which is the uncertainty of the measurement across the length of the step. The derivation of *u_Lstep_* in Eq. (35) is given in this section.

Recall that the step height as obtained from one step height test structure is given by Eq. (12) and repeated below:

(191) $$stepN_{XY}=\frac{stepN_{XYa}+stepN_{XYb}+stepN_{XYc}}{3}$$

This equation can be written in terms of the platform measurements by using Eq. (8) to obtain the following equation:

(192) $$\begin{array}{l} stepN_{XY}=\frac{1}{3}[(platNYa-platNXa)\\ +(platNYb-platNXb)+(platNYc-platNXc)]. \end{array}$$

Rearranging, this equation becomes:

(193) $$\begin{array}{l} stepN_{XY}=\frac{1}{3}[(platNYa+platNYb+platNYc)\\ -(platNXa+platNXb+platNXc)]. \end{array}$$

Defining *platNX*_ave_ and *platNY*_ave_ as the average of the platform height measurements from *platNX* and *platNY*, respectively, this equation can be rewritten as follows:

(194) $$stepN_{XY}=platNY_{\text{ave}}-platNX_{\text{ave}}.$$

Given the above equation, the uncertainty equation for *u_Lstep_* can be written as follows:

(195) $$u_{Lstep}=\sqrt{u_{LpNX}^{2}+u_{LpNY}^{2}},$$

where *u_LpNX_* is the uncertainty across the length of *platNX* and where *u_LpNY_* is the uncertainty across the length of *platNY*.

The uncertainty equation for *u_LpNX_* is determined from estimated minimum and maximum step height values (namely, *stepN_XY_*_min_ and *stepN_XY_*_max_, respectively) as given below:

(196) $$\begin{array}{r} stepN_{XY\min}=platNY_{\text{ave}}-[platNX_{\text{ave}}\\ +3(\sigma_{platNX\text{ave}}-\sigma_{\text{rough}NX})] \end{array}$$

and

(197) $$\begin{array}{r} stepN_{XY\max}=platNY_{\text{ave}}-[platNX_{\text{ave}}\\ -3(\sigma_{platNX\text{ave}}-\sigma_{\text{rough}NX})], \end{array}$$

where *σ_platNX_*_ave_ and *σ*_rough_*_NX_* are defined in Sec. 4.3 and also in Sec. 9.2. With 99.7 % confidence, assuming a Gaussian distribution (and assuming *u_LpNY_* and all other sources of uncertainty equal zero), the value for *stepN_XY_* lies between *stepN_XY_*_min_ and *stepN_XY_*_max_. Therefore, *u_LpNX_* is calculated as follows:

(198) $$u_{LpNX}=\frac{stepN_{XY\max}-stepN_{XY\min}}{6},$$

which [using Eq. (196) and Eq. (197)] simplifies to

(199) $$u_{LpNX}=\sigma_{platNX\text{ave}}-\sigma_{\text{rough}NX},$$

which is comparable to Eq. (165) in Sec. 9.2 for one platform. Similarly, the equation for *u_LpNY_* is as follows:

(200) $$u_{LpNY}=\sigma_{platNY\text{ave}}-\sigma_{\text{rough}NY}.$$

Inserting Eq. (199) and Eq. (200) into Eq. (195) produces the following equation for *u_Lstep_*:

(201) $$u_{Lstep}=\sqrt{{\left(\sigma_{platNX\text{ave}}-\sigma_{\text{rough}NX}\right)}^{2}+{\left(\sigma_{platNY\text{ave}}-\sigma_{\text{rough}NY}\right)}^{2}},$$

which is Eq. (35) in Sec. 4.3. This model for *u_Lstep_* was chosen because if *σ_platNX_*_ave_ = *σ*_rough_*_NX_* = 0 and *σ_platNY_*_ave_ = *σ*_rough_*_NY_*, then *u_Lstep_* = 0.
